# Histidine-rich enantiomeric peptide coacervates enhance antigen sequestration and presentation to T cells†

**DOI:** 10.1039/d5sc01163a

**Published:** 2025-03-25

**Authors:** Ushasi Pramanik, Anirban Das, Elise M. Brown, Heather L. Struckman, Huihao Wang, Samuel Stealey, Macy L. Sprunger, Abdul Wasim, Jonathan Fascetti, Jagannath Mondal, Jonathan R. Silva, Silviya P. Zustiak, Meredith E. Jackrel, Jai S. Rudra

**Affiliations:** a Department of Biomedical Engineering, McKelvey School of Engineering, Washington University in St. Louis St. Louis MO 63130 USA srudra22@wustl.edu; b Department of Chemistry, Washington University in St. Louis St. Louis MO 63130 USA; c Department of Biomedical Engineering, Saint Louis University St. Louis MO 63103 USA; d Tata Institute of Fundamental Research Hyderabad Hyderabad 500046 India

## Abstract

Peptides and peptidomimetics that self-assemble through LLPS have recently emerged as vital building blocks for creating functional biomaterials, thanks to their unique physicochemical properties and dynamic nature. One of life's most distinctive features is its selectivity for chiral molecules. To date, coacervates comprised of d-amino acids have not been reported. Here, we demonstrate that histidine-rich repeats of (GHGXY)_4_ (X = L/V/P) and their enantiomers undergo LLPS, paving the way for improved coacervate stability. Through a series of biophysical studies, we found that the droplet size can be tuned based on L, V, or P substitution, and molecular cargo between 600 and 150 000 Da is efficiently recruited in a bioactivity-preserving aqueous environment during phase separation. Mechanistic studies reveal that the droplets enter cells *via* energy-dependent endocytic pathways, exhibit composition-selective fusion properties, and effectively deliver molecular therapeutics across various cell types. Finally, we demonstrate that the coacervates enhance antigen presentation to CD4^+^ and CD8^+^ T cells, resulting in robust proliferation and the production of functional cytokines. Our study outlines the development and characterization of enantiomeric peptide coacervates as promising vaccine delivery vehicles with tunable physicochemical properties.

## Abstract 2

Chiral histidine-rich peptides form coacervates that improve antigen delivery, T cell proliferation, and functional cytokine production.

## Graphical abstract

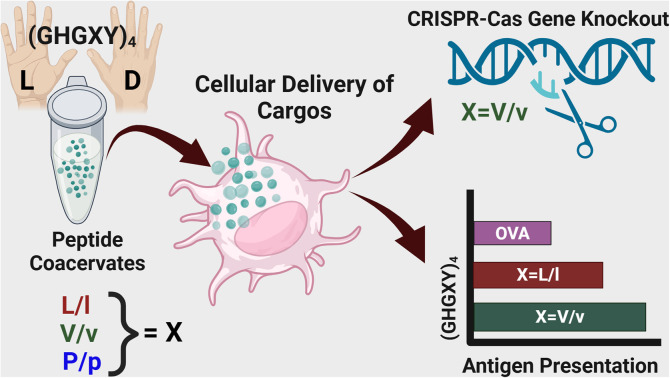

## Introduction

Liquid–liquid phase separation (LLPS) is a phenomenon in which associative interactions between macromolecules and segregative interactions with the external environment result in the formation of solute-rich coacervates or droplets.^1–5^ LLPS enhances the spatiotemporal separation and concentration of essential cellular components, facilitating processes like gene expression, proliferation, and differentiation.^6,7^ Intrinsically disordered proteins (IDPs) are particularly prone to LLPS. However, many natural and synthetic short peptides can also form simple or complex coacervates and serve as tools to elucidate the determinants of LLPS processes.^8–12^

Peptides serve as appealing building blocks for creating LLPS coacervates because of the chemical diversity inherent in amino acids. This diversity can enable the adjustment of key features that trigger phase separation, which can also be adjusted to fine-tune the bulk properties of the resulting droplets. By adjusting the primary sequences of peptides, one can modify their charge, hydrophobicity, and chirality to create ideal conditions for both the assembly and disassembly of coacervates, thereby enabling control over the spatiotemporal release of the loaded cargo.^13–16^ Unlike polymeric or inorganic nanocarriers that require organic solvents,^17^ linkers,^18^ and complex conjugation chemistry,^19^ droplets can form spontaneously in bioactivity-preserving aqueous buffers, making them attractive for *in vivo* applications. Due to their dynamic nature, phase-separating droplets can undergo interactions with cell membranes, leading to efficient transport of cargo into cells with minimal toxicity.^20^ These advantages make peptide-based coacervates appealing functional biomaterials for the cellular delivery of therapeutics.^21–24^ Several LLPS systems based on natural and synthetic peptides and other block polymers have been described for applications as scaffolds for regenerative medicine and tissue engineering, bioactive drug delivery, and functional probes for disease diagnosis and therapy.^22–26^

In recent years, there has been an emerging interest in the introduction of chirality into biomaterials.^27^ Chirality, inherent to all life processes, drives most biochemical reactions in organisms through selectivity for chiral molecular species (*e.g.*, l-amino acids and d-sugars).^28,29^ Owing to the natural use of l-amino acids, the fundamental change in backbone-side chain connectivity of D-enantiomers makes them resistant to proteases and immune recognition.^30,31^ It remains unclear how the substitution with d-amino acids affects the kinetics of phase separation, droplet size, encapsulation efficiency, environmental sensitivity, and stability, presenting a new avenue for research. Miserez and co-workers have pioneered the development of phase-separating histidine-rich protein domains (HBPs) or peptides (HBpeps) derived from the beaks of jumbo squid, demonstrating that they undergo LLPS to form coacervates, and have explored their potential application in biomedicine.^28^ Similar to VPGXG repeats in elastin, HBpeps contain GHGXY repeats, where a minimum of four repeats are required for coacervate formation, with X mostly being Leu (L), Val (V) or Proline (P).^32^ Histidine and tyrosine residues are critical for the phase separation of HBpeps, which is driven by the H-bonding of deprotonated histidine with tyrosine residues and proceeds in physiological buffers without the need for crowding agents.^33^ The short length of HBpeps and ease of production using synthetic chemistry facilitates the rational study of a range of constructs.

Here, we utilized LLPS-prone histidine-rich pentapeptide repeats of (GHGXY)_4_ (where X = L/I/F/V/A/P/N) to assess how hydrophobicity and chirality impact coacervate formation. Our data show that (GHGXY)_4_ peptides and their enantiomers exhibit identical LLPS properties that depend on concentration, pH, and ionic strength. Replacing X with L/V/P resulted in different coacervate sizes of approximately 1.8 μm, 1 μm, and 0.2 μm, respectively. We also tested the encapsulation efficiency of various cargo (600 Da–150 kDa) and performed rheological studies to assess viscoelastic behavior. Mechanistic studies demonstrated that the droplets entered cells *via* energy-dependent endocytic pathways, and microscopy data indicated that leucine droplets fused with other droplets as well as with cell membranes, while valine droplets resisted fusion. Notably, coacervates of d-amino acids also led to increased and prolonged antigen presentation compared to their natural counterparts. Thus, our study demonstrates that LLPS is not modulated by chirality and exemplifies chiral phase-separating systems as appealing vaccine delivery vehicles with tunable physicochemical properties.

## Results and discussion

### Phase separation of histidine-rich repeat peptides

**Fig. 1:**
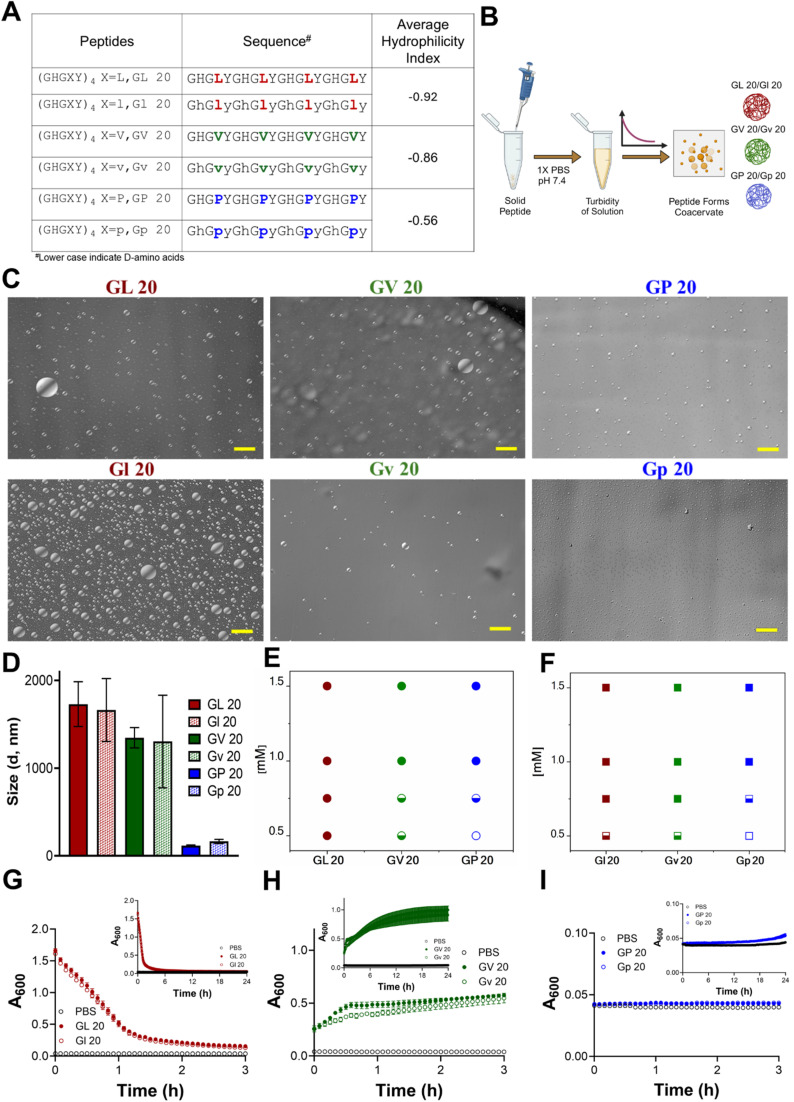
Chiral (GHGXY)_4_ sequences and their LLPS properties. (A) The sequence of l- and d-peptides and their average hydrophilicity index (AHI). The AHI index values were calculated using the online peptide calculator. (B) Schematic of l- and d-peptide dissolution, followed by optical microscopy imaging. (C) Optical micrographs of chiral (GHGXY)_4_ peptides (scale: 50 μm). (D) DLS analysis shows size variation between l- and d-(GHGLY)_4_, (GHGVY)_4,_ and (GHGPY)_4_ (L > V > P). Data is an average (mean ± SEM) of 3 technical replicates with 14 scans for each measurement. (E and F) Phase diagram of l- and d-(GHGXY)_4_ peptides at different concentrations. Filled circles/squares: complete coacervate formation; open circles/squares: no coacervate formation; half-filled circles: low coacervate formation. Turbidity (*λ* = 600 nm) measured over time (3 h) for (G) GL 20, (H) GV 20, (I) GP 20. The inset shows the full kinetics up to 24 h. Filled circles: l-peptides; open circles: d-peptides. Grey circles represent the control (1× PBS).

To investigate the role of hydrophobicity in the formation of HBpep coacervates, we introduced amino acids with varying hydrophobicity indices (F > L ∼ I > V > P > A > N) at the X position (Fig. S1†). The data indicated that hydrophobicity was a crucial factor in coacervate formation, as no droplets were detected for (GHGAY)_4_ and (GHGNY)_4_, while (GHGFY)_4_ resulted in a viscous gel. Consistent coacervate formation across multiple synthesis batches was observed for (GHGLY)_4_, (GHGIY)_4_, (GHGVY)_4_, and (GHGPY)_4_. Given the isomeric nature of leucine and isoleucine, we focused on the characterization and application of coacervates where X = L/V/P. Furthermore, modulation of chirality presents a subtle change, as enantiomeric peptides are identical in length, side-chain size, flexibility, hydropathy, charge, and polarity, yet allow for manipulation of specific biological outcomes. In this study, we generated all-L or all-D amino acid variants of the (GHGXY)_4_ peptide, where (X = L/V/P), analyzed their coacervate properties and their utility for antigen delivery to T cells. The sequences of all peptides are shown in Fig. 1A with lowercase denoting d-amino acids. The identity and purity of the peptide variants were confirmed using MALDI-TOF (Fig. S2–S4†) and HPLC (Fig. S5–S7†).

### Chirality does not impact droplet characteristics or LLPS kinetics

The average hydrophilicity index (AHI) (https://www.bachem.com/knowledge-center/peptide-calculator/) was calculated for the variants, and a negative value is indicative of their hydrophobic nature (L > V > P). Phase separation was evaluated using optical microscopy and showed that no coacervates were formed in pure water or salt solutions (150 mM NaCl) (Fig. S8 and S9†). The peptides formed coacervates only in physiological buffers (1× PBS, pH 7.4) (Fig. 1B and C), signifying that the phosphate ions in PBS play an important role in the coacervation. DLS analysis showed that droplet sizes varied with hydrophilicity. Still, it was identical for each pair of enantiomers and ranged from ∼1.8 μm for (GHGLY)_4_, ∼1.0 μm for (GHGVY)_4_, and ∼0.2 μm for (GHGPY)_4_ (Fig. 1D). Prior studies using GHGXY analogs with multiple-length repeats, variants, and substitutions have enhanced our understanding that hydrophobic interactions play a significant role in coacervation.^34^ Substituting a GAGFA repeat in HBpeps also led to dense hydrogel formation instead of liquid coacervates, presumably due to enhanced hydrophobicity and stronger intermolecular interactions.^33,34^ Based on these observations, the critical concentration (*C*_crit_) of coacervate formation was assessed. Data indicated that *C*_crit_ values decreased as the hydrophobicity of the amino acid at the X position increased (*L* = 0.5 mM, *V* = 0.75 mM, *P* = 1 mM) (Fig. 1E and F), and as expected, the enantiomeric variants displayed identical *C*_crit_ values (Fig. S10 and Table S1†).

LLPS is promoted by increased solvent entropy resulting from peptide desolvation and increased peptide conformational freedom.^35^ The rapid movement of peptides inside the droplets leads to maturation and coalescence. The kinetics of LLPS were measured by observing the turbidity of the solution at 600 nm. We observed divergent kinetics of coacervate formation and maturation for the different variants. Solutions of (GHGLY)_4_ formed a turbid solution instantaneously, as evidenced by the high *A*_600_ value at *t* = 0 (Fig. 1G), which decayed with time, likely reflecting the droplets' rapid maturation and coalescence.^12,36,37^ A striking difference in turbidity was observed for (GHGVY)_4_, which steadily increased over 24 h (Fig. 1H). Interestingly, no turbidity changes were detected with time for the (GHGPY)_4_ peptide, suggesting that the initial formation of droplets observed upon its instant dissolution may be the rate-limiting step for coacervate formation (Fig. 1I).^37^ This difference in kinetics is likely due to hydrophobicity and inter- and intramolecular interactions that drive coacervate formation.^36,38,39^ We propose that (GHGLY)_4_ has a greater propensity to form droplets instantaneously due to its more hydrophobic nature, contributing to its rapid coalescence to reduce interfacial tension and a swift decrease in turbidity measurements. Because (GHGVY)_4_ is less hydrophobic than (GHGLY)_4_, we observed a steady increase in the turbidity, which can be a signature of slower droplet formation. As expected, all enantiomers exhibited kinetically identical profiles of LLPS (Fig. 1G–I).

To further exclude the possibility that the drop in turbidity for the leucine peptides could be due to the settling of coacervates, turbidity measurements were made with or without agitation. Data indicated no differences between the two conditions (Fig. S11†). An acknowledged caveat with using turbidity assays to describe the kinetics of LLPS is that plate readers are not fast enough to resolve early nucleation and growth events in droplet formation.^37^ To enhance rigor and align with LLPS reports, turbidity measurements were also carried out at 350 nm with no detectable differences (Fig. S12†). Overall, the distinct differences in the kinetics of (GHGXY)_4_ variants illustrate that amino acid hydrophobicity (L > V > P) significantly influences coacervate formation and maturation.

### The effects of temperature, salt concentration, and pH on coacervate formation

External factors, such as pH and ionic strength, are significant in phase separation through charge screening and ‘salting out’ effects. To evaluate this, we examined the effect of salt concentration on coacervate formation. All variants formed droplets across a wide range of NaCl concentrations (Fig. S13†) but only within a narrow range of pH values (Fig. S14†). The highest relative turbidity was noted at 150 mM NaCl and pH 7.4. This is not surprising since the peptides have a calculated isoelectric point of approximately 7.66, where electrostatic repulsions are minimized, promoting coacervation.^40^ Optical micrographs of coacervates under various salt and pH conditions are shown in Fig. S11D and S12D,† respectively. The data confirmed the presence of coacervates in leucine and valine variants even at 1 M NaCl, while no coacervates were detected for the proline variant at concentrations exceeding 250 mM NaCl (Fig. S13†).

A complete understanding of the effects of salt on LLPS remains an outstanding challenge as phase separation depends on both the salt concentration and the chemical identity of ions, which is called the Hofmeister effect.^41–43^ Wu *et al.* tested the effects of different ions of the Hofmeister series on HBpeps and reported a broader two-phase region in the more kosmotropic sodium sulfate (Na_2_SO_4_) compared to sodium chloride (NaCl). The more chaotropic sodium bromide (NaBr) disrupted coacervation and allowed phase separation to occur only at higher peptide concentrations.^34^ It has been proposed that the competition between the solvation energy and translational entropy of the ion determines solubility at high salt concentrations.^41^ At higher salt concentrations, increasing hydrophobicity resulted in a significant decrease in *C*_crit_ (L > V > P) with a broader two-phase region for leucine peptides (Fig. S15A and D†). In contrast, enantiomers of valine (Fig. S15B and E†) and proline (Fig. S15C and F†) showed a comparatively narrow two-phase region. This is presumably due to the shielding effect on protonated histidine residues, with reduced double-layer repulsion and entropy loss for counter-ion balancing, which in turn lowers *C*_crit_ for all variants.^44,45^

To assess the differences in temperature-dependent LLPS behavior of (GHGXY)_4_ peptides, turbidity measurements were conducted at room temperature (RT) and 37 °C. The turbidity of the leucine peptide solutions decayed slightly faster at 37 °C than at RT, likely due to increased solubility. We detected very subtle differences in turbidity for all peptides and their enantiomers at 37 °C compared to RT (Fig. S16†). This is promising, as these properties could enable the encapsulation, retention, and delivery of therapeutics without significant concerns of *in vivo* stability.

### Secondary structures of peptide coacervates

**Fig. 2:**
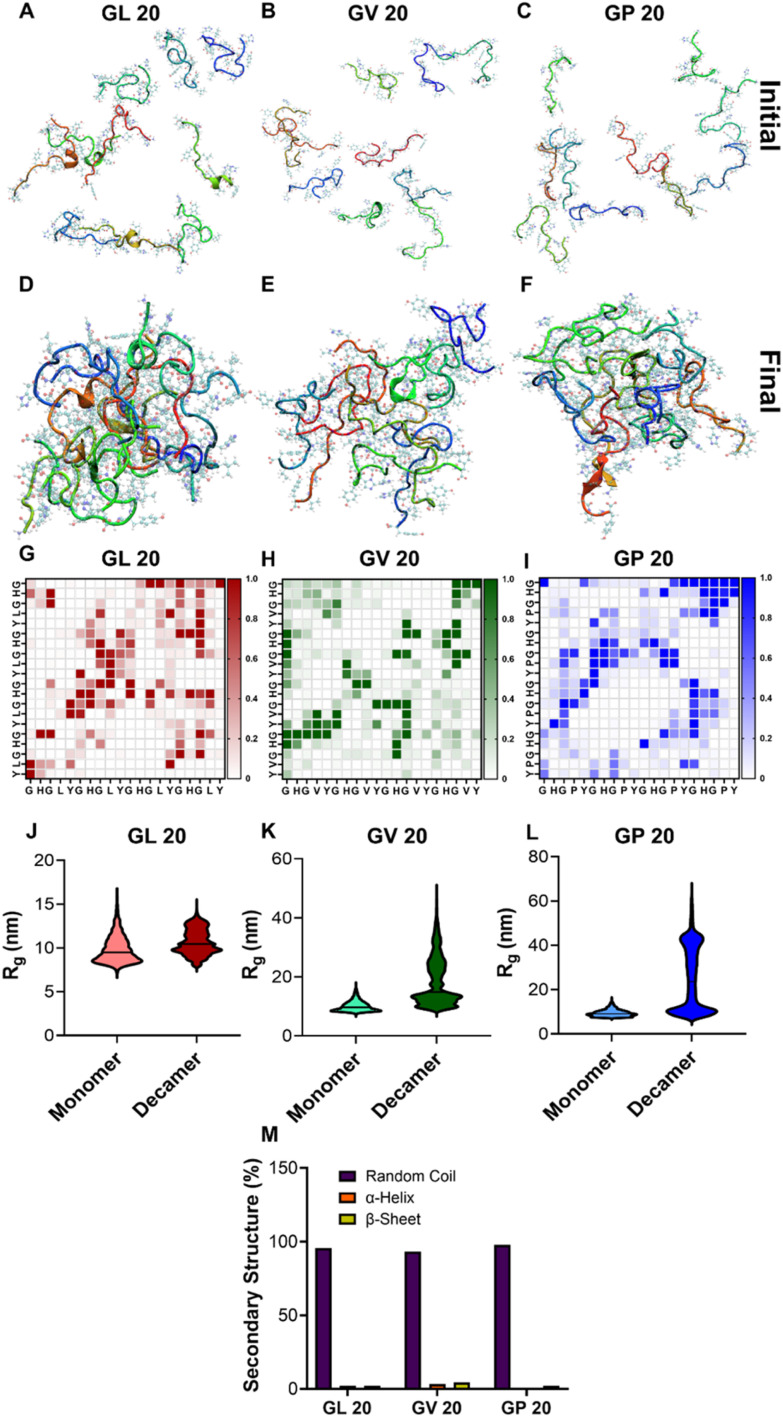
All-atom simulations illustrate molecular interactions in LLPS by (GHGXY)_4_ peptides. Initial and final snapshots of multichain all-atom simulations for (A and D) GL 20, (B and E) GV 20, and (C and F) GP 20, using 10 monomeric peptides for each within a box. The contact map for (G) GL 20, (H) GV 20, and (I) GP 20 is generated by merging all chains in the simulation box into a single droplet. This contact map offers an overview of the molecular interactions within each chain that drive and stabilize LLPS by these peptides. The color scale ranges from lowest (0, white) to highest (1, color) contact probability between the amino acids of two such peptides (horizontal and vertical axes). *R*_g_ plots confirm LLPS for the monomer and decamer of (J) GL 20, (K) GV 20, and (L) GP 20. (M) The percentage of secondary structure is calculated from all-atom simulations for (GHGXY)_4_ peptides.

We next probed the secondary structures of (GHGXY)_4_ variants using CD and FT-IR spectroscopy. The CD profiles of all peptides depicted ellipticity centered between 225 and 230 nm, attributed to π–π* transitions arising from aromatic interactions, also known as the cotton effect (Fig. S17A–C†).^46,47^ Moreover, we observed a red-shift in ellipticity maximum from 228 nm (GHGLY)_4_ to 230 nm (GHGVY)_4_ to 232 nm (GHGPY)_4_, consistent with a decrease in hydrophobicity from L > V > P, known as the positive cotton effect.^47^ The CD spectra of all enantiomers were mirror images of each other, as expected. The FT-IR spectra of the variants showed subtle differences in their secondary structures (Fig. S18†). The amide-I bands of leucine and valine peptides were centered around 1643 and 1647 cm^−1^, respectively. The valine peptide had an additional band at 1634 cm^−1^. In contrast, the proline variant had bands centered at 1628 and 1637 cm^−1^, which typically represent β-sheet structures. However, none of the peptides exhibited β-sheet fibril formation and all-atom molecular simulations found minimal to no β-sheet content in all variants (Fig. 2M).

### Mechanical properties of peptide coacervates

It is now widely recognized that biomolecular coacervates can be viscoelastic, and studies on IDP domains have reported both shear-thinning and shear-thickening behavior.^48,49^ We assessed the mechanical properties of freshly prepared coacervate suspensions (droplets and dilute phase together) using parallel plate rheology at a fixed shear rate (Fig. S19A†). Data indicated that their viscosity was dependent on peptide hydrophobicity (L > V > P), where we observed leucine droplets exhibited greater viscosity compared to valine or proline variants (Fig. S19B†). Increasing the peptide concentration from 1 to 2 mM increased viscosity for all peptides (Fig. S19B†). Prior reports on LLPS by various peptides have reported an environment-dependent shear-thinning or shear-thickening behavior, and increasing the shear rate led to a decrease in viscosity with no differences observed between the enantiomers (Fig. S19C†). Such shear-thinning behavior may be advantageous for using coacervates as injectable biomaterials. Further, we measured the storage and loss moduli (*G*′ and *G*′′, respectively, Fig. S20†) of the coacervate solutions, and the ratio of *G*′/*G*′′ > 1 indicated a viscoelastic nature. Leucine coacervates exhibited nearly a 3-fold increase in *G*′ compared to valine droplets, whereas a 2-fold increase in *G*′ values was noted for valine compared to proline. Rheological studies on minimal HBpep sequences have highlighted that the viscoelastic properties of the coacervates could be tuned by single residue mutations, which can, in turn, alter the secondary structure content.^34^ While no secondary structures have been observed for the (GHGXY)_4_ variants, their mechanical properties strongly correlated with hydrophobicity, and our data support prior findings that the viscoelastic properties of coacervates can be fine-tuned by minor alterations in the peptides' primary sequences.

### Deciphering molecular interactions by all-atom simulations

We conducted multi-chain, all-atom simulations of (GHGXY)_4_ variants to understand the molecular interactions driving coacervate formation. We initiated the simulations from a dispersed state (Fig. 2A–C) and observed a single droplet forming for each variant during the simulations (Fig. 2D–F). Next, we examined each peptide's average inter-peptide contact probabilities (Fig. 2G–I). Each contact probability was calculated as a time average of the residue–residue contacts between any two chains and then averaged over the number of peptide pairs. This contact map was generated after all the chains in the simulation box had merged into a single droplet, providing an overview of the molecular interactions within each chain that drive and stabilize coacervate formation. We observed that the pattern in the contact map varied among different peptides despite 80% sequence homology. This demonstrates that even a single residue change in the repeating unit of the sequence can lead to vastly different interactions driving LLPS, potentially contributing to distinct rheological and physical properties of the phase-separated droplets. Notably, all sequences showed high contact probabilities between histidine residues, although the differences in histidine–tyrosine interactions varied widely among peptide sequences. This suggests that (GHGXY)_4_ peptides tend to form coacervates through strong and frequent cation–π and π–π interactions, along with the nature of the side chain also playing an important role in coacervate formation.

To quantify these interactions, we calculated the number of π–π interactions during the last 500 ns of the simulation trajectory, a duration during which all chains have formed a single, stable droplet. We calculated the radius of gyration (*R*_g_) of individual chains under two scenarios: (i) a single chain in a box of water and (ii) a chain in a droplet. We then plotted the corresponding *R*_g_ values for both cases (Fig. 2J–L) and observed an increase in *R*_g_ for scenario (ii), indicating LLPS.^50,51^ Next, we characterized the π–π interactions that drive droplet formation; the data in Fig. S21A–F† provides relative snapshots of parallel and perpendicular π-stacking interactions from the simulation trajectories. We also compared the number of parallel and perpendicular π-stacking interactions that occurred during the last 500 ns of simulation trajectories for all three peptides. (GHGPY)_4_ showed the highest number of parallel π-stacking interactions, followed by (GHGLY)_4_ and (GHGVY)_4_ (Fig. S21G†). A similar trend was observed for perpendicular π-stacking interactions (P > L > V). These findings support data in Fig. 2G–I panels, where (GHGPY)_4_ showed a high contact probability between H and Y, compared to L or V variants.

### Encapsulation efficiency and cellular uptake of coacervates

**Fig. 3:**
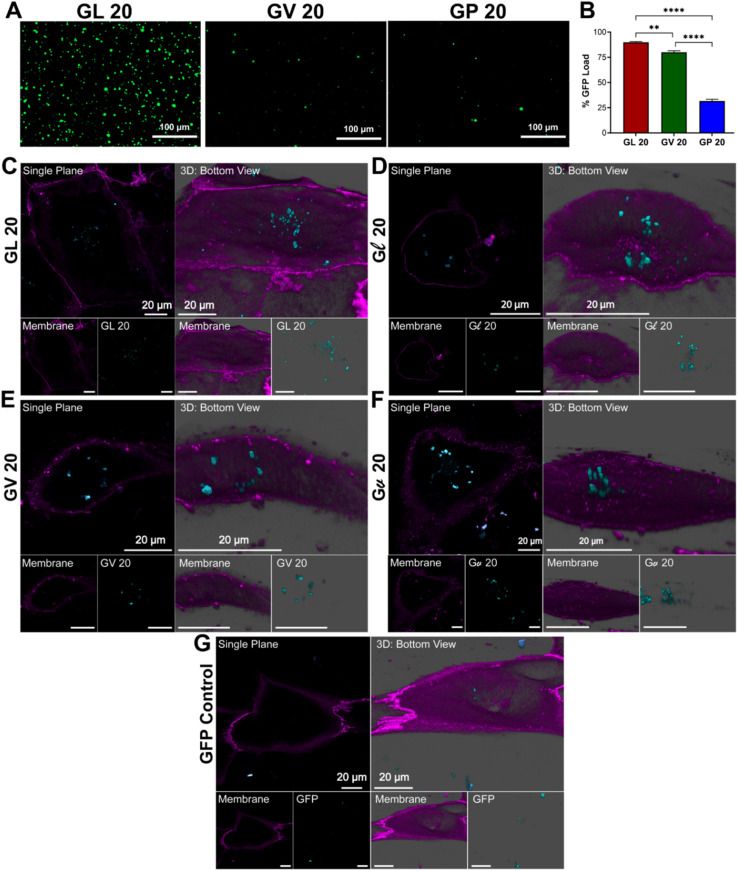
Localization of GL 20, Gl 20, GV 20, Gv 20 within hiPSC-CMs. Fluorescence microscopy images of eGFP loaded (A) GL 20, GV 20, and GP 20 coacervates (scale bar: 100 μm). (B) % eGFP load by GL 20, GV 20, and GP 20 droplets. **p < 0.01, ****p < 0.0001 as determined by a one-way ANOVA. Representative confocal images from hiPSC-CMs treated with (C) GL 20, (D) Gl 20, (E) GV 20, (F) Gv 20, (G) eGFP control (teal). The cellular membrane was identified with WGA membrane stain (purple). Micrographs were presented as single plane and 3D snapshots from the bottom view perspective (scale bars: 20 μm).

We next tested the capacity of (GHGXY)_4_ coacervates to encapsulate cargo for cellular delivery. The encapsulation efficiency was calculated from absorbance or fluorescence measurements of the cargo in the dilute and the condensed phases and confirmed using microscopy (Fig. 3A and S22†). Using eGFP, we calculated the loading efficiency to be the highest for the leucine droplets (∼90%) compared to valine (∼75%) or proline (∼30%) coacervates (Fig. 3B). A wide range of cargo (600 Da–150 kDa) was tested, and data confirmed that encapsulation efficiency varied with hydrophobicity (L > V > P) (Fig. S23†). Our findings differ from reports by the Lampel lab, wherein the most hydrophobic peptide droplets had the lowest GFP encapsulation efficiency. In contrast, the most polar peptide droplets entrapped the greatest quantity of GFP.^38^ A plausible explanation can be that the number of π–π, cation–π, or hydrogen bonding interactions between the peptide and cargo could dictate the loading capacity, thereby explaining the differences in encapsulation between the peptides.^38^ Due to their small size and poor encapsulation efficiency, (GHGPY)_4_ droplets were not tested further.

To further illustrate the utility of these coacervates as delivery vehicles across various cell lines, we used eGFP loaded (GHGLY)_4_ and (GHGVY)_4_ coacervates and confocal microscopy to visualize cellular uptake in hiPSC-CM (human induced pluripotent stem cell-derived cardiomyocyte) cultures. After 30 min of incubation, single-plane bottom views confirmed their localization within the cells (Fig. 3C–F). The images show that both leucine and valine coacervates (teal) are fully encapsulated by the membrane (purple) of the hiPSC-CMs and are centrally positioned. Qualitatively, the coacervates enhanced delivery efficiency compared to the soluble eGFP control (Fig. 3G). Single plane top view images were also acquired to verify the intracellular localization of the droplets (Fig. S24†). No significant differences were noted between the internalization of the enantiomeric variants. These results demonstrate that the encapsulation efficiency of (GHGXY)_4_ coacervates can be tuned by the primary sequence.

### The coalescence properties of coacervates

Similar to water, the fusion of biomolecular coacervates into larger droplets has been reported for various membraneless organelles, including nucleoli, stress granules, and Cajal bodies.^52^ Dynamic coacervates can undergo rapid fusion despite the high macroscopic viscosity inside them. Given the differences in hydrophobicity, size, viscoelasticity, and encapsulation efficiency among the coacervates, we next tested their fluidity by capturing video still images over time (Fig. S25A and B†). The data indicated multiple rapid fusion events for the leucine coacervates only which aligns with the LLPS kinetics data in Fig. 1G, where a rapid decay in turbidity was attributed to droplet fusion (video in Fig. S25†). However, the droplet edges did not fully resolve, suggesting arrested fusion mechanisms.^53,54^ As an additional readout, individual leucine coacervates loaded with red, blue, or green fluorophore-tagged proteins were mixed (red: PE-Cy7-mouse IL-4; blue: EF 450-mouse IFN-γ; green: FITC mouse anti-mouse H-2K^b^) and coalescence was observed over time (Fig. S25C†). Microscopy data showed droplets with secondary colors (fusion of two primary colors) or grey (fusion of three primary colors), suggesting droplet fusion (Fig. S25D†), after about 15 minutes of mixing. In comparison, valine droplets did not exhibit fusion behavior (Fig. S25G and H†). To test whether the coacervates can encapsulate multiple cargo molecules without exclusion or compositional drift, we mixed RBG fluorophore-tagged proteins prior to loading (Fig. S25E and I†). Data showed that the bulk of the leucine and valine droplets encapsulated all three proteins with few coacervates showing only binary mixtures (Fig. S25F and J†). Control experiments with mixed protein solutions without coacervates were used to validate encapsulation within the droplets (Fig. S26†).

At the interface of the coacervate-bulk phase, the surface molecules experience a net force toward the interior of the bulk phase. This interfacial tension drives the fusion of two encountering liquid-like coacervates. A recent study by Sun *et al.* reported that fluid-like or gel-like coacervates can be achieved *via* systematic hydrophobic or charged amino acid mutations in GHGXY repeats.^55^ The study also found differences in uptake rates and intracellular release kinetics of cargo where fluid-like droplets exhibited enhanced cell membrane adhesion and wetting compared to gel-like coacervates.^55^ Our data suggests that the leucine coacervates have a low to moderate interfacial tension and undergo fusion readily compared to the valine droplets, thereby decreasing its interfacial area through droplet coalescence.^39^ Using confocal microscopy, we also observed that leucine coacervates interacted with and traversed the cell membrane (orange dashed circle, Fig. S27A and B† left panel). The same droplet was found to be deformed and stretched, spanning the intercellular membrane and extracellular spaces as shown in the orthogonal projection (right side *Z* plane panel) (Fig. S27C†). Also, intracellular leucine coacervates contained membrane signals (yellow dashed circle) indicating coacervate–lipid interactions. The co-localization of membrane signal (purple) with coacervates (teal) in the intracellular space of hiPSC-CMs is shown in Fig. S27B.† These observations suggest that the leucine droplets cross the cell membrane, and these events may contribute to their internalization. Despite our best efforts, this behavior was not observed for valine coacervates.

We next evaluated whether the leucine and valine coacervates could effectively deliver molecular therapeutics using eGFP plasmid DNA in HEK293 cells or CRISPR/Cas9 ribonucleoprotein (RNP) complexes in A549 cells. The data demonstrated robust eGFP expression in HEK293 cells treated with the leucine droplets (Fig. S28A and B†). Qualitatively, the delivery efficiency was lower than lipofectamine 2000 but higher than the naked plasmid. Poor eGFP expression was detected in cells treated with valine coacervates. Our gene editing complex consisted of the Cas9 protein and the guide RNA (sgRNA) targeting the knockdown of the CD55 gene. Data indicated that enantiomeric coacervates of (GHGVY)_4_ successfully delivered the RNP complex, resulting in a corresponding decrease in CD55 expression on the cell surface, as measured by flow cytometry (Fig. S28C†). In contrast, (GHGLY)_4_ coacervates were not highly effective. However, the delivery efficiency for both the peptide coacervates was lower than that of lipofectamine, as reported by Miserez and co-workers.^56^ The coacervates showed no cytotoxicity under the experimental conditions tested (Fig. S29 and S30†).

### The mechanisms of cellular internalization of coacervates

**Fig. 4:**
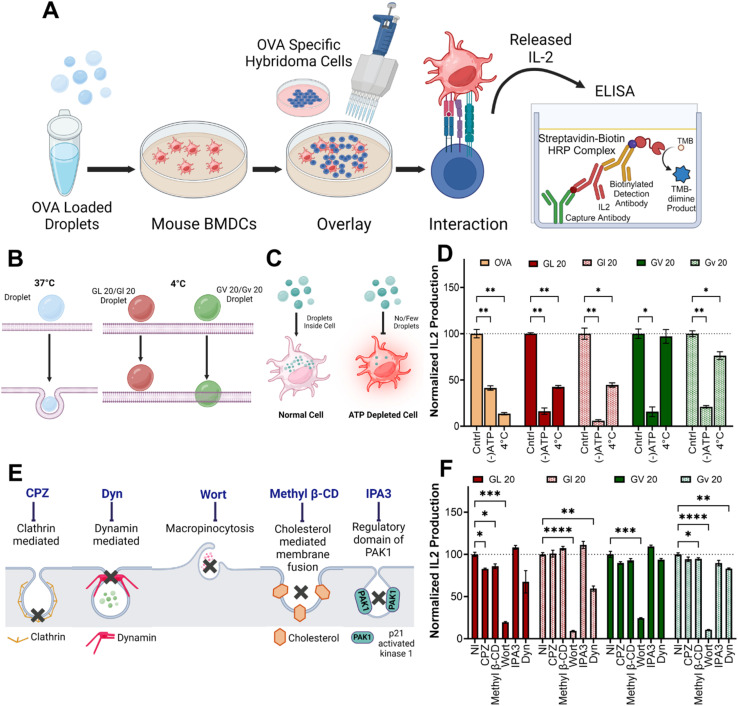
Mechanisms of cellular uptake studies. (A) Schematic of *in vitro* antigen presentation assay depicting IL-2 production by hybridoma cells overlaid on BMDCs treated with OVA-loaded coacervates. (B and C) Schematic representation of OVA loaded L or D coacervates in ATP-depleted cells at 4 °C and 37 °C. (D) IL-2 production by OVA-loaded L or D coacervates upon ATP depletion, and at 4 °C, normalized to 37 °C (Cntrl). (E) Schematic of different endocytosis inhibitors and their target pathways. (F) Effect of endocytosis inhibitors on the uptake of L or D (GHGXY)_4_ (L/V) coacervates loaded with OVA. A pronounced inhibitory effect was observed for Wortmannin (Wort), which inhibits macropinocytosis. *p < 0.05, **p < 0.01, ***p < 0.001, ****p < 0.0001 as determined by one-way ANOVA.

To test internalization mechanisms, we utilized *in vitro* antigen presentation assays (Fig. 4A). Here, bone marrow-derived dendritic cells (BMDCs) are treated with coacervates loaded with the model antigen ovalbumin (OVA). Following incubation, BMDCs are washed extensively to remove extracellular coacervates and overlaid with hybridomas that specifically recognize the OVA_323–339_ peptide in the context of MHC class II (DOBW cells) or the OVA_257–264_ peptide in the context of MHC class I (CD8 OVA 1.3 cells). Secretion of IL-2 in response to peptide-MHC recognition is an indirect measure of coacervate uptake and can be quantified using ELISA (enzyme-linked immunosorbent assay) (Fig. 4A). In DCs treated with OVA-loaded (GHGLY)_4_ and (GHGVY)_4_ coacervates at 4 °C and overlaid with DOBW cells, significantly lower IL-2 levels were detected compared to 37 °C controls (Fig. 4B and D). To further confirm that the differences in uptake were not due to reduced plasma membrane fluidity, we used ATP-depleted media at 37 °C to block all energy-dependent pathways (Fig. 4C).^57^ Again, a significant loss of IL-2 secretion was observed, suggesting that the droplets are internalized through energy-dependent mechanisms (Fig. 4D). A key advantage of this assay compared to flow cytometry is that it eliminates positive readouts from membrane-bound coacervates and requires complete internalization and processing of OVA. We next used pharmacological inhibitors of endocytic mechanisms to explore mechanisms of cell entry (Fig. 4E). BMDCs were treated with inhibitors of clathrin-mediated endocytosis (chlorpromazine, CPZ),^58^ dynamin-dependent endocytosis (dynasore, Dyn),^59^ macropinocytosis (wortmannin, Wort),^60^ cholesterol (methyl β-CD),^61^ and IPA3 the regulatory domain of PAK1 (IPA-3)^62^ for one h prior to antigen presentation assays. Treatment with wortmannin had the most significant effect on uptake of coacervates, indicating that the formation of macropinosomes likely plays an important role in their endocytosis. In contrast, CPZ, which disrupts clathrin-mediated endocytosis; the GTPase inhibitor Dyn, which inhibits dynamin activity; and methyl β-CD, which disrupts cholesterol-mediated lipid rafting, did not significantly impact droplet uptake (Fig. 4F). This is similar to what was observed by Miserez and co-workers, who observed an energy-dependent micropinocytosis mechanism as the process by which the HBpep and HBpep-SP peptides are internalized by cells.^63^

### Activation of innate immunity by coacervates

Understanding their ability to activate innate immune cells is crucial for the ongoing development of peptide coacervates as vaccine delivery vehicles. BMDCs are key regulators of adaptive immunity with the potential to induce T cell activation/immunity or T cell suppression/tolerance. Previous studies have demonstrated that peptide biomaterials can exert adjuvant-like effects by inducing the upregulation of maturation markers such as MHC-II, CD80, and CD86, along with the secretion of chemokines (MCP-1α/CCL2, KC/CXCL1) and cytokines (GM-CSF, IL-5, IL-6, IL-1β).^64,65^ To test this, we treated BMDCs with (GHGLY)_4_ and (GHGVY)_4_ coacervates or LPS as positive control and assessed cytokine and chemokine production using multiplex assays. Data indicated that the coacervates were immunologically inert and did not induce the production of pro-inflammatory (IL-6 and TNF-α), tolerance-inducing (IL-10), or maturation-specific (IL-15) cytokines (Fig. S31†). We simultaneously profiled the expression of inflammatory chemokines, specifically monocyte chemoattractant protein-1 (MCP-1), macrophage inflammatory proteins (MIP-1α/MIP-1β), and IFN-γ-inducible protein 10 (IP-10) which are essential for mounting an effective immune response due to their ability to recruit other immune cells (Fig. S31†). No chemokines or growth factors (GM-CSF, EGF, MIF, and PGDF-BB) that modulate immune cell proliferation or host immune responses were detected, suggesting that the coacervates do not activate innate immune signaling. These purely structural scaffolds can now be suitably modified with select immune agonists that activate pathways specific to conferring protection against infections, non-infectious diseases, autoimmune disorders, or allergies.

### Antigen delivery, processing, and presentation mechanisms

**Fig. 5:**
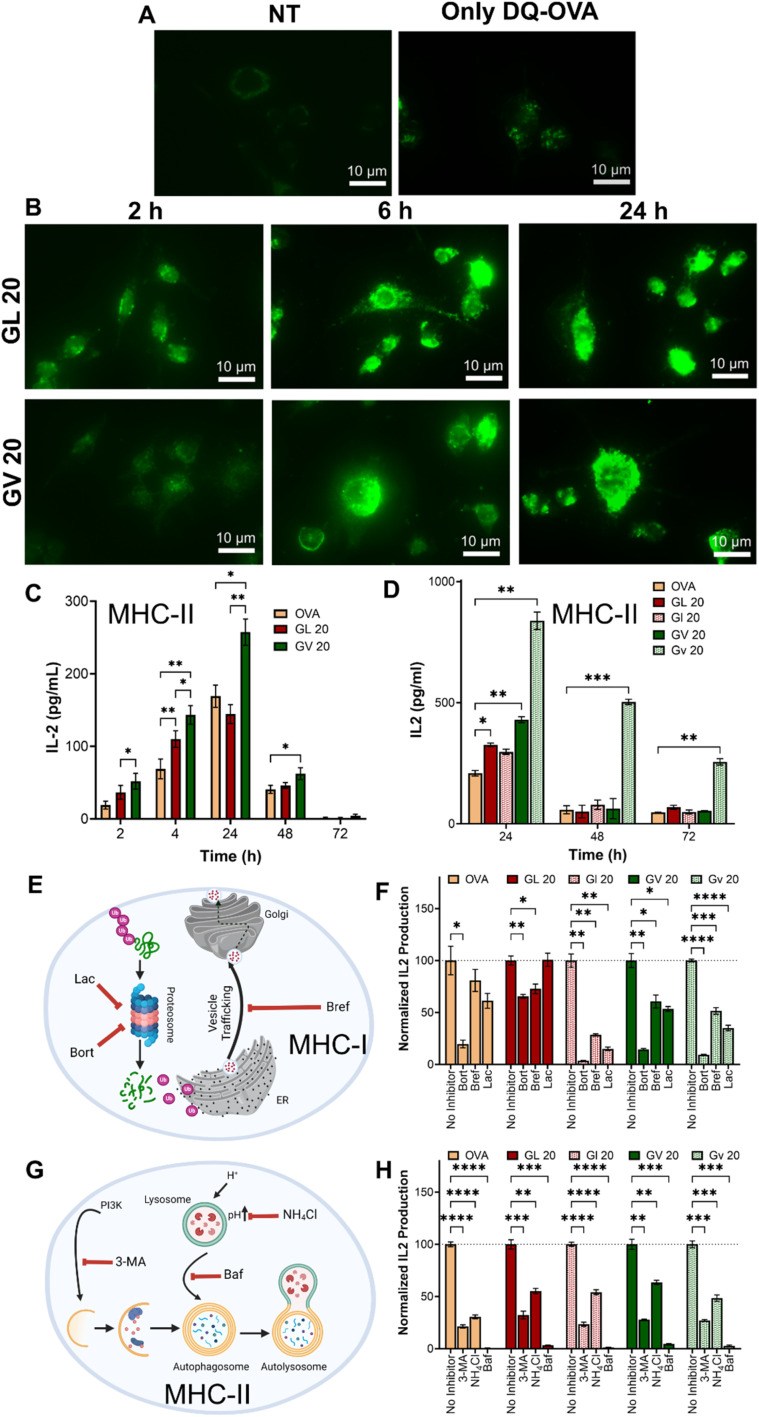
Kinetics of antigen presentation and antigen presentation pathways in BMDCs treated with OVA-loaded coacervates. (A) DQ-OVA fluorescence in mouse BMDCs treated with saline or soluble DQ-OVA compared to (B) DQ-OVA delivered using GL 20 or GV 20 coacervates. (C) Comparison of antigen presentation between GL 20 and GV 20 coacervates loaded with 1 μM OVA as a function of time compared to soluble OVA. (D) Comparison of antigen presentation kinetics between GL 20 or GV 20 coacervates and their enantiomers. All coacervates were loaded with 10 μM OVA. (E and G) Schematics illustrating the inhibition of MHC-I and MHC-II antigen presentation pathways. (F) Reduced IL-2 production in the presence of MHC I inhibitors (bortezomib, brefeldin A, and lactacystin) or (G) MHC-II inhibitors (3-MA, NH_4_Cl, and bafilomycin) following delivery of OVA using enantiomeric GL 20 or GV 20 coacervates. *p < 0.05, **p < 0.01, ***p < 0.001, ****p < 0.0001, as determined by one-way ANOVA.

Dendritic cells scan peripheral tissues for antigens, which, once acquired, are carried to the lymph nodes, where they induce adaptive immune responses. This requires the lysosomal processing and breakdown of the antigens into peptide fragments that can be presented to T cells. A number of polymeric carriers exploiting lysosomal delivery and lysosomal escape have been reported for the induction of effective immune responses.^66^ To evaluate the ability of the coacervates to deliver antigens to lysosomal compartments, we loaded (GHGLY)_4_ and (GHGVY)_4_ with DQ-OVA, which, upon protease-mediated hydrolysis in the lysosomes, produces fluorescent peptides. Green fluorescent puncta were detected in DCs treated with both coacervates, indicating lysosomal processing of DQ-OVA (Fig. 5A and B and S32†). To quantify delivery, we pre-treated DCs with the coacervates for 2 h, 4 h, 24 h, 48 h, or 72 h before hybridoma overlay and measured IL-2 levels in the supernatant (Fig. 5C). Compared to soluble OVA, the coacervates enhanced antigen presentation as evidenced by increased IL-2 production up to 24 h. IL-2 production steadily decreased with time at 48 h and was not detectable at 72 h, suggesting antigen clearance. Importantly, for the same antigen dose, IL-2 production was significantly higher in DCs treated with (GHGVY)_4_ droplets compared to (GHGLY)_4_ (Fig. 5C). Further, a comparison between enantiomers indicated a prolonged antigen presentation by D-form (GHGVY)_4_ coacervates with robust IL-2 signal detectable even at 72 h compared to the L-form (Fig. 5D). Surprisingly, this effect was absent in cells treated with D-form GL 20 coacervates. A potential explanation here is the merging of leucine droplets that combine to form larger droplets, while valine droplets do not. Some cargo may be lost during the droplet fusion process and those only that successfully enter the cells will release their contents, enabling functional activity. This type of instability due to droplet fusion has previously been highlighted by Webber and co-workers.^36^ We deduce that the stability of valine droplets may explain the improved antigen delivery and IL-2 production. The amount of antigen processed and presented depends on protecting the cargo and ensuring optimal antigen delivery into the lysosomal compartments. Because of their instability, the leucine droplets are inherently poor at delivering antigens. Additionally, loading OVA into d-amino acid coacervates offers extra stability against serum and cellular proteases. Thus, the effect observed here results from using valine droplets with higher stability and low degradability. Taken together, these findings indicate that chiral peptide coacervates are excellent carriers for enhancing antigen delivery and prolonged presentation for vaccine development applications. We also confirmed our findings observed with the model antigen OVA, using Ag85B, an immunodominant protein from *Mycobacterium tuberculosis* and BB7 hybridoma cells, which recognize Ag85B_240–254_ in the context of MHC class II (Fig. S33†).

Classically, exogenous antigens internalized by DCs are processed in the lysosomal compartments and presented *via* MHC-II to CD4^+^T cells, whereas cytosolic antigens processed *via* the proteasomal pathway and presented *via* MHC-I to CD8^+^T cells. Non-canonical presentation of exogenous antigens on MHC-I and cytosolic antigens on MHC-II has been reported to provide protection against certain viral infections and cancers. We next tested whether antigens delivered using peptide coacervates are processed *via* classical MHC-I (Fig. 5E) or MHC-II pathways (Fig. 5G). DCs were treated with the three different inhibitors of each pathway 30 min prior to the addition of coacervates, and IL-2 secretion by overlaid DOBW or OVA1.3 hybridoma cells was used to assess MHC-II or MHC-I antigen presentation, respectively. At the tested concentrations, none of the inhibitors were significantly cytotoxic (Fig. S34†). A significant decrease in IL-2 production was observed in DCs pre-treated with bafilomycin, NH_4_Cl, or 3-methyl adenine (3-MA), which are well-known MHC-II inhibitors (Fig. 5H). Bafilomycin specifically inhibits V-ATPase, preventing the acidification of endosomes and lysosomes, while NH_4_Cl raises the pH of intracellular compartments, thereby disrupting the processing of endocytosed materials. Lastly, 3-MA is an autophagy inhibitor that blocks class III PI3K activity. Further, the fluorescence intensity in DCs treated with DQ-OVA coacervates was reduced in the presence of NH_4_Cl or bafilomycin, indicating decreased proteolytic activity (Fig. S35†). Similarly, MHC class I presentation was significantly diminished as evidenced by lower IL-2 secretion in all groups treated with bortezomib, lactacystin, or brefeldin A. Bortezomib and lactacystin are proteasomal inhibitors, while brefeldin A inhibits the trafficking of degraded peptides from the endoplasmic reticulum for MHC class I presentation (Fig. 5F). These findings suggest that antigens delivered using peptide coacervates are processed and presented through classical MHC pathways.

Overall, the enhanced antigen presentation using D-form GV 20 coacervates is highly encouraging for the development of vaccines and immunotherapies. However, biomaterials fabricated *via* LLPS have only been recently reported, and their stability *in vivo* has yet to be evaluated. Also, while using D-form peptides may be advantageous for improving delivery and potentially reducing the amount of antigen needed in vaccines, researchers have reported increased accumulation due to slower clearance *in vivo*.^67^ This could potentially lead to long-term toxicity in applications where repeated administration may be required. Thus, the concentration of d-amino-based coacervates *in vivo* has to be carefully adjusted for their usage as delivery platforms for various biomolecules.

### Induction of functional T cell responses by coacervates

While hybridoma cell lines are valuable as tools to quantify antigen presentation and elucidate mechanisms, T cell proliferation assays assess the ability of antigens to stimulate T cells to divide and produce effector cytokines as activation indicators. We utilized CD4^+^T and CD8^+^T cells isolated from transgenic mice that specifically recognize the OVA_323–339_ and OVA_257–264_ epitopes in the context of MHC class II or MHC class I molecules, respectively. Purified T cells were labeled with a fluorescent dye and overlaid onto DCs treated with OVA-loaded coacervates. Proliferation was quantified based on the fluorescence intensity of the daughter cells, and cytokine production was assessed using multiplex assays (Fig. S36A†). Our data demonstrated robust proliferation of both CD4^+^T and CD8^+^T cells with no significant differences between groups or enantiomers for CD4^+^T cells (Fig. S36B†). However, the proliferation in the (GHGLY)_4_ group was slightly higher than the (GHGVY)_4_ group. Similarly robust proliferation was detected in CD8^+^ T cells with a higher percentage of proliferated cells in the (GHGVY)_4_ group compared to the (GHGLY)_4_ group (Fig. S36G†). A significant difference was also noted between (GHGLY)_4_ enantiomers. To determine the nature of the T cell response, we measured the cytokine profile in the supernatants. Data indicated robust levels of Th1 (IFN-γ and IL-2) and Th17 (IL-17) cytokine production by OT-II and OT-I T cells compared to Th2 (IL-4, IL-5) cytokines (Fig. S36C–F, H–K and S37–S40†). Th1 cytokines, particularly IFN-γ, are proinflammatory and essential for controlling intracellular viral infections, while Th17 cytokines (*e.g.*, IL-17A) mediate host defensive mechanisms, especially against extracellular bacterial infections. These results pave the way for future work to establish the efficacy of coacervates as effective vaccine delivery vehicles for combating bacterial or viral infections.

## Conclusions

In conclusion, we have demonstrated the development and testing of enantiomeric peptide coacervates as biomaterials for delivering and presenting antigens to T cells. The d-amino acid coacervates discussed in this study represent a significant advancement not only in creating novel materials but also as model systems to enhance our understanding of the role of peptide and protein coacervates in health and disease. The intermolecular and intramolecular interactions among peptides, the interfacial tension and fusion, and the bulk properties of the resulting droplets can be adjusted through simple amino acid substitutions in the primary sequence. Research on cargo encapsulation and cellular uptake mechanisms confirm that chirality is not necessary for the application of coacervates as delivery vehicles in biomedical contexts. The ability to enhance delivery to cytosolic or endosomal compartments based on the primary sequence allows for control over desired biological functions. The inert characteristics of the coacervates, along with their capacity to improve antigen presentation, offer opportunities for further modification with immunogenic or tolerogenic signals to regulate the immune response. Due to the spontaneous formation of coacervates in a bioactivity-preserving physiological environment and the efficiency of antigen delivery, they present substantial potential for developing vaccines and immunotherapies targeting chronic infectious and non-infectious diseases.

## Author contributions

Conceptualization, U. P., A. D., M. E. J. and J. S. R.; methodology, J. M., J. R. S., S. P. Z., M. E. J., J. S. R.; investigation, U. P., A. D., E. M. B., H. L. S., H. W., S. S., M. L. S., A. W., J. F.; writing – original draft, U. P., A. D., and J. S. R.; writing – review & editing, J. M., J. R. S., S. P. Z., M. E. J., J. S. R.; funding acquisition, J. M., M. E. J., J. S. R.; resources, J. M., J. R. S., S. P. Z., M. E. J., J. S. R.; supervision, J. S. R.

## Conflicts of interest

There are no conflicts to declare.

## Supplementary Material

SC-016-D5SC01163A-s001

SC-016-D5SC01163A-s002

## Data Availability

All data generated from this study are available upon request.
