# Supplementary material for: Histidine-rich enantiomeric peptide coacervates enhance antigen sequestration and presentation to T cells
Source: Chem Sci. 2025 Mar 25;16(17):7523–36. doi: 10.1039/d5sc01163a (PMC11955804; doi:10.1039/d5sc01163a)
Supplement: SC-016-D5SC01163A-s001 [file SC-016-D5SC01163A-s001.pdf]

Supplementary Information (SI) for Chemical Science.
This journal is © The Royal Society of Chemistry 2025

Supporting Information

Histidine-rich enantiomeric peptide coacervates enhance antigen sequestration and presentation to T cells

Ushasi Pramanik[#,a], Anirban Das[#,a,b], Elise M. Brown[a], Heather L. Struckman[a], Huihao Wang[a], Samuel Stealey[c], Macy L. Sprunger[b], Abdul Wasim[d], Jonathan Fascetti[a], Jagannath Mondal[d], Jonathan R. Silva[a], Silviya P. Zustiak[c], Meredith E. Jackrel[b], and Jai S. Rudra[a,*]

[a]Department of Biomedical Engineering, McKelvey School of Engineering, Washington University in St. Louis, St. Louis, MO, 63130 USA

[b]Department of Chemistry, Washington University in St. Louis, St. Louis, MO, 63130 USA

[c]Department of Biomedical Engineering, Saint Louis University, St. Louis, MO, 63103 USA

[d]Tata Institute of Fundamental Research Hyderabad, Hyderabad 500046, India

[*]Correspondence: Jai S. Rudra, srudra22@wustl.edu

[#]These authors contributed equally.

Materials and Methods..S4-S16
Figure S1. Peptide sequences and their optical images S17
Figure S2. MALDI-TOF spectra of L-$(GHGXY)_4$ peptides (L/V/P) S18
Figure S3. MALDI-TOF spectra of D-$(GHGXY)_4$ peptides (l/v/p) **...** S19
Figure S4. MALDI-TOF spectra of L-$(GHGXY)_4$ peptides (A/F/I/N) **....................................** S20
Figure S5. HPLC analysis of L-$(GHGXY)_4$ peptides (L/V/P).. S21
Figure S6. HPLC analysis of D-$(GHGXY)_4$ peptides.. S22
Figure S7. HPLC analysis of L-$(GHGXY)_4$ peptides (A/F/I/N)... S23
Figure S8. LLPS of $(GHGXY)_4$ peptides in water... S24
Figure S9. LLPS of $(GHGXY)_4$ peptides in 150 mM NaCl S25
Figure S10. Determination of C_{crit} for LLPS S26
Table S1: C_{crit} for L- and D-peptides S26
Figure S11. Kinetics of LLPS at A_{600} without shaking... S27
Figure S12. Kinetics of LLPS at A_{350} S28
Figure S13. Effect of NaCl concentration on coacervation of $(GHGXY)_4$ peptides S29
Figure S14. Effect of pH on coacervation of $(GHGXY)_4$ peptides... S30
Figure S15. Critical phase separation concentrations of $(GHGXY)_4$ variants S31
Figure S16. Effect of temperature on LLPS kinetics S32
Figure S17. CD profiles of L- and D-peptides... S33
Figure S18. FT-IR spectra of L- and D-peptides S34
Figure S19. Mechanical testing of peptide coacervates.. S35
Figure S20. Storage and loss modulus of $(GHGXY)_4$ peptide solutions.................................. S36
Figure S21. Parallel and perpendicular π-stacking interactions from all-atom simulations...... S37
Figure S22. Microscopy images of eGFP loaded $(GHGXY)_4$ droplets S38
Figure S23. Encapsulation efficiency of $(GHGXY)_4$ droplets.. S39
Figure S24. 3-D Top view images of GFP loaded coacervates in hiPSCs S40
Figure S25. Representative coalescence images of $(GHGLY)_4$ droplets and cargo encapsulations. S41
Figure S26. Controls depicting fluorescence in the absence of coacervates S42
Figure S27. Membrane interactions of GL 20 in hiPSC-CMs.. S43
Figure S28. Delivery of plasmid DNA or Cas9 RNPs (protein + gRNA)................................ S44

Figure S29. Cytotoxicity of coacervates in HEK293T cell line S45
Figure S30. Cytotoxicity of coacervates in primary murine BMDCs....................................... S46
Figure S31. Cytokine production by BMDCs following coacervate treatment......................... S47
Figure S32. DQ-OVA fluorescence in BMDCs at various time points.................................... S48
Figure S33. Antigen presentation using coacervates S49
Figure S34. Cytotoxicity of inhibitors S50
Figure S35. DQ-OVA fluorescence intensity in the presence of inhibitors.............................. S51
Figure S36. OT-I/OT-II mouse $CD8^+$/$CD4^+$ T cell proliferation.. S52
Figure S37. Flow data for transgenic $CD4^+$T cells S53
Figure S38. Cytokine production by transgenic $CD4^+$T cells.. S54
Figure S39. Flow data for transgenic $CD8^+$T cells S55
Figure S40. Cytokine production by transgenic $CD8^+$T cells.. S56
References..S57-S58

MATERIALS AND METHODS

Peptide synthesis and purification

The L- and D-enantiomers of $(GHGLY)_4$, $(GHGVY)_4$ and $(GHGPY)_4$ peptides were purchased from GenScript and used without further modification. $(GHGAY)_4$, $(GHGFY)_4$, $(GHGIY)_4$ and $(GHGNY)_4$ were synthesized using standard Fmoc-SPPS chemistry on Rink Amide with Oxyma (Ethyl cyano(hydroxyimino) acetate) and N, N′-diisopropylcarbodiimide (DIC) as coupling agents on a Liberty Blue microwave-assisted synthesizer. Peptides were cleaved using a cocktail of trifluoroacetic acid (TFA), tri-isopropyl silane (TIS), and H_2O (95:2.5:2.5) and extracted and washed in cold diethyl ether. The resulting pellet was dissolved in acetonitrile/water mixture (50:50) and frozen prior to lyophilization. Peptides were purified (~90%) using High Performance Liquid Chromatography (HPLC) on a Dionex Ultimate 3000 HPLC equipped with a diode-array detector on an Agilent Poroshell RP-C18 column (4.6 mm×150 mm), operating at a flow rate of 1 mL/min. Peaks corresponding to 220 nm were collected for MALDI-TOF mass analysis (Shimadzu MALDI-8030) using α-cyno-4-hydroxycinnamic acid matrix (Bruker Daltonics, MA).

Turbidity assays

Peptide solutions (0.25–1.5 mM) were prepared in 1×PBS at pH 7.4. Aliquots (140 µL) were pipetted into a 96-well plate and absorbance at 600 nm was measured over 24 h using a Synergy HT plate reader (Biotek, USA) at RT or 37°C with or without agitation. The pH dependence of LLPS formation was assessed in 0.1 M potassium phosphate buffer (with 150 mM NaCl) at different pH values (5.8, 6.6, 7.4, 8.0) and 1 mM peptide concentration. Similarly, 0.1 M potassium phosphate buffer (pH 7.4) with varying salt concentrations (0, 150, 250, 500, 1000 mM

NaCl) was used to study ionic strength effects. The absorbance was further converted to turbidity using methods previously described [1] and data was plotted as relative turbidity.

Optical microscopy and dynamic light scattering (DLS)

Droplets were visualized in 1×PBS using NCI Leica DMC4500 microscope in reflection mode with differential interference contrast. Images were captured with a DMC4500 camera under the control of Leica LAS X software (version 3.4.2). Coacervate size was measured using 1 mM peptide solutions (1×PBS, pH 7.4) on a ZEN 3600 Zetasizer (Malvern Instruments, UK) using DLS-Zen 0040 specialized cuvettes. The results were reported as the means of three measurements, with 14 scans for each measurement.

Transmission electron microscopy (TEM)

Peptide solutions prepared for TEM were applied directly to 200-mesh, carbon-coated copper grids for 2 min and stained with 1% uranyl formate for 1 min. Excess stain was blotted using filter paper. Brightfield images were taken with a JEOL JEM-1400 transmission electron microscope with a NANOSPRT15 camera at an accelerating voltage of 120 kV.

Rheology testing

Viscosity of the peptide solutions (1 mM or 2 mM) was tested using an ARES 2000ex rotational rheometer (TA Instruments, New Castle, DE). Peptides were freshly solubilized, and 170 µL of each was pipetted onto the rheometer stage. A 20-mm parallel plate geometry was lowered to a gap of 200 µm. Storage modulus (G′) and loss modulus (G″) were measured as a function of angular frequency (1–10 rad/s) with 1% strain. Viscosity was measured as a function

of shear rate (1–100 s^{-1}). Separately, viscosity was measured using a Hagen-Poiseuille viscometer (RheoSense microVISC, San Ramon, CA) at a shear rate of 2500 s^{-1} to confirm viscosity differences at two different concentrations (1 mM and 2 mM).

Computational simulations

All simulations were performed using GROMACS 2023 [2,3] and the CHARMM36m [4] force field. The necessary force field parameters and input files were generated using AlphaFold [5] predicted structures coupled with CHARMM-GUI [6]. A single peptide chain was solvated in a 5-nm cubic box. Sodium and chloride ions were added to maintain electroneutrality and achieve 0.15 M NaCl. The system was energy minimized using the steepest descent algorithm and then underwent 125 ps of NVT equilibration followed by 125 ps of NPT equilibration, with 0.001 ps time steps. During these equilibration steps, the peptide atom coordinates were restrained to prevent changes. The system was further equilibrated for 125 ps in the NPT ensemble without restraints and with a 0.002 ps time step. The final equilibrated system was used for production runs with a 0.002 ps time step. Different configurations were extracted from single-chain simulations and used to build the initial multi-chain system. Ten chains, each obtained from a distinct time point or simulation of a single monomeric peptide, were incorporated into a 10-nm cubic box. Peptides were solvated and electro-neutralized, and 0.15 mM NaCl was added to achieve physiological conditions. Energy minimization was performed on the resulting system.

Next, 125 ps of NVT equilibration followed by 125 ps of NPT equilibration, with 0.001 ps time steps and no position restraints, was carried out on the energy-minimized system. The final equilibrated conformation was used for production runs with a 0.002 ps time step. For all simulations, coordinates were saved after every 1,000 steps. The V-rescale thermostat and C-

rescale barostat were used for all simulations with standard temperature and pressure coupling parameters. π-π stacking interactions were calculated using a 0.5 nm distance cutoff between the centers of mass of the aromatic rings. For each pair of aromatic residues, the distance between their sidechain centers of mass was measured. If this distance was ≤ 0.5 nm and if the angle between the plane of the aromatic rings was not between 30°–60°, the pair was engaged in a π-π stacking interaction. Additionally, angles between the aromatic rings were analyzed to classify the interactions as either parallel stacking (0°–30°) or perpendicular stacking (60°–90°).

Fourier-transform infrared (FT-IR) spectroscopy

FT-IR measurements for secondary structural analysis were conducted using 1 mM peptide solutions (1×PBS, pH 7.4) with a Bruker Alpha II FT-IR instrument equipped with a Smart Performer single-reflection ATR accessory and an Au crystal sample stage. The background spectrum and buffer spectrum were collected and subtracted from the sample using OPUS software. An average of 24 scans for each peptide were employed for each sample measurement. Data were analyzed using GRAMS/AI software (Thermo Scientific, USA). Second derivative spectra were calculated from the absorbance spectra in the Amide I region using a Savitzky-Golay filter, third order, with a nine-point window. Analyzed second derivative spectra between 1610 cm^{-1} and 1710 cm^{-1} were fit with six or seven Gaussian curves, informed by the Akaike information criterion, and the peak positions were compared to literature reports [7–9].

Circular Dichroism (CD) spectroscopy

The CD spectra of the peptide solutions (0.75 mM) were recorded on a Jasco J-815 CD spectrometer. The spectra (average of three scans for each sample) were collected within the

wavelength range of 215–260 nm with a bandwidth of 1.00 nm, 0.5 nm step. Solvent background was subtracted from each spectrum and data converted to mean residue ellipticity (MRE).

Droplet coalescence assays

Experiments were conducted to visualize the dynamic process of droplet fusion by capturing a series of still images over 15 min, with each acquisition every 500 ms. Contrast and brightness were adjusted to ensure optimal visibility of the coacervates, and noise cancellation was applied to reduce background interference. Frame correction aligned the images to account for shifts that occurred during capture. Additional enhancements included sharpening to visualize finer details and color correction to ensure that the visual representation closely matched the actual observation. The images were meticulously stacked to create a continuous video sequence that provided a dynamic view of the peptides' behavior, showing the evolution of coacervates from their initial formation, through stabilization and fusion. Significant fusion events were identified and marked with arrows. Advanced tracking algorithms were employed to follow movement and changes within the coacervates, and event segmentation was used to isolate specific interactions of interest.

Stem cell-derived cardiomyocytes and confocal microscopy

Wild type hiPSCs (WT iPSC 11) were received from the Genome Engineering and Stem Cell Center at Washington University School of Medicine. hiPSCs were cultured in mTeSR Plus medium (STEMCELL Technologies, USA) on 6-well plates coated with Matrigel (1:100, Corning) and grown at 37ºC with 5% CO_2. Cells were grown to 80–90% confluency and then passaged as clusters using Versene (Thermo Scientific, USA) into 6-well plates for hiPSC maintenance and

into 24-well plates for hiPSC differentiation. Once hiPSCs reached 90% confluence, they were differentiated into cardiomyocytes using small-molecule manipulation of Wnt signaling [10]. Non-cardiomyocytes were removed via lactate purification on days 20 and 22 before replating singularized hiPSC cardiomyocytes (hiPSC-CMs) at 40,000 cells per 35-mm glass-bottom dish.

hiPSC-CMs were treated with enantiomeric leucine or valine coacervates loaded with eGFP protein (Novus Biologicals, Catalog number: NBP2-34923; 50 μL, final concentration of 100 nM eGFP) for 30 min at 37°C. Samples were fixed with 2% paraformaldehyde (PFA) and three PBS washes (10 min at RT). The cell membrane was stained with wheat germ agglutinin (WGA; Fisher Scientific, Catalog number: W32466; 0.25 μL/mL) conjugated to Alexa Fluor 647 (10 min at RT), followed by three PBS washes (5 minutes at RT). Confocal micrographs of singular hiPSC-CMs were acquired using a Leica Sp8 Lightning single-photon confocal microscope equipped with five solid-state lasers (405, 488, 514, 552, and 638 nm, 12 mW each), a 63×/1.4 numerical aperture oil immersion objective, two HyD GaAsP detectors, and two high-sensitivity photomultiplier tube detectors. Images were collected as z-stacks (presented as top or bottom view, and single plane) and by Nyquist sampling (or greater) as previously described [11].

BMDC cultures

All animal experiments were conducted under approved protocols by the Institutional Animal Care and Use Committee (IACUC) at Washington University in St Louis. Femoral and tibial bone marrow from C57BL/6 mice were collected and RBCs lysed using ACK buffer. The cells were plated at 5×10^6 cells per petri dish (150 mm) and differentiated for 7–9 days in complete RPMI-1640 medium (containing 10% heat-inactivated FBS), 55 μM β-mercaptoethanol, 1 mM sodium pyruvate, 10 mM HEPES, 1× MEM non-essential amino acids, 20 ng/mL GM-CSF, 10

ng/mL IL-4, and 100 μg/mL penicillin-streptomycin (pen-strep). Non-adherent cells were collected by pipetting and washing plates gently with media and plated at required density.

Encapsulation efficiency measurements

Stock solutions of eGFP (248 μM), FITC-H-2K^b antibody (BD Biosciences, Catalog number: BDB553569; 0.5 mg/mL), or BODIPY labeled ovalbumin (DQ-OVA, Catalog number: D12053, Thermo Fisher Scientific; 1 mg/mL) were solubilized in PBS and added to dry peptide powders to a final peptide concentration of 1 mM. Cargo concentrations in the stock solutions were 1 μM eGFP, 50 μg/mL FITC- H-2K^b, and 1 mg/mL DQ-OVA. Samples were then centrifuged at 15,000 × g for 10 min, and the absorbance of the supernatant was measured for BODIPY (488 nm) and fluorescence for eGFP (λ_{ex}: 395 nm and λ_{em}: 510 nm) and FITC (λ_{ex}: 495 nm and λ_{em}: 519 nm). For small molecule dyes, similar methods were used. The encapsulation efficiency was determined as reported previously [12].

Cellular uptake and antigen presentation assays

Uptake of coacervates by BMDCs was measured using *in vitro* antigen presentation assays. BMDCs were plated in 96-well round-bottom plates (1×10^5 cells/well) and maintained at 4°C or treated with 10 mM deoxy glucose and 10 mM sodium azide in PBS (ATP depletion) for 1 h prior to addition of OVA-loaded coacervates. Final OVA concentration was 1 μg/mL for time-dependent measurements and 10 μg/mL for comparison between enantiomers. Cells maintained at 37°C and complete RPMI media served as controls. Soluble OVA and PBS treated cells were used as controls for antigen delivery. Following treatment (2 h, 4 h, 24 h, 48 h, or 72 h), BMDCs were washed to remove extracellular coacervates and DOBW hybridoma cells (1:5, DC: DOBW) were

overlaid for 16 h. The cells were then centrifuged at 300 × *g* for 5 min, and the supernatant was collected for IL-2 quantitation by ELISA according to the manufacturer protocol (Biotechne, #DY402). The amount of IL-2 produced under each condition was measured. A second antigen/hybridoma pair (Ag85B protein and BB7 hybridoma)[13] was used to confirm that the findings were not OVA-specific. Final antigen concentration was ~0.5 μM and the ratio of BMDCs to BB7 cells was 1:7. Inhibition of coacervate uptake was measured by adding the inhibitors 1 h prior to coacervate treatment for 24 h followed by hybridoma overlay and IL-2 measurements. DOBW and OVA1.3 hybridoma cells were a kind gift from Dr. Clifford V. Harding (Case Western Reserve University) [14,15]. BB7 hybridoma was a kind gift from Dr. David Canaday (Case Western Reserve University) [16].

Cytotoxicity and transfection assays

For cytotoxicity assays, HEK293T cells or primary mouse BMDCs (5×10^4 cells/mL in 96-well plates) were treated with peptide coacervates (100 μM) for 24 h. MTT (3-(4, 5-dimethylthiazolyl-2)-2, 5-diphenyltetrazolium bromide (Sigma-Aldrich)) solution (40 μL of 1 mg/mL stock diluted in media) was added to each well and cells were incubated further for 24 h. Media was then removed, and 150 μL DMSO was added to each well. Absorbance was measured at 570 nm using a BioTek Synergy H1 microplate reader. Untreated cells or ethanol treated cells served as controls. Cytotoxicity of all inhibitors was evaluated similarly. For transfection studies, HEK293T cells (1×10^4 cells/mL in 24-well plates) were allowed to adhere and grow (24 h) prior to treatment with $(GHGLY)_4$ or $(GHGVY)_4$ coacervates loaded with eGFP DNA plasmid (Altogen Biosystems, Catalog number: 4060; 2.5 μg/mL). Cells treated with the naked plasmid or

complexed with lipofectamine 2000 (3.2 μL) served as controls. GFP expression was assessed qualitatively after 96 h using a Lionheart FX automated microscope (BioTek Instruments, Inc.).

CRISPR-Cas Assay

We employed TrueCut Cas9 protein for the knockout of CD55 gene using CRISPR technology, aided with chiral $(GHGXY)_4$ peptide coacervates. The transfection of the Cas9 RNPs (protein + gRNA) was done according to manufacturer's protocol (Invitrogen). Briefly, A549 cells were cultured in complete DMEM media (supplemented with 10% FBS and 5% Pen Strep) in T-75 flask till full confluency. Cells were then plated at a concentration of 2×10^4 cells/mL in 24 well plates in DMEM media for 24 h. On the day of transfection, the cells were observed to be ~50 % confluent. For positive control, we mixed the TrueCut™ Cas9 protein/sgRNA in Opti-MEM media (17.8 μL) at a concentration of 12.50 μg (75 pmol; Stock 0.5 mg/mL) and 2.4 μg (75 pmol; Stock 1 nmol) for protein and sgRNA respectively, containing 50 μL Lipofectamine™ Cas 9 Plus™ reagent (Total Volume: 100 μL) in a RNAse-free microcentrifuge tube. In another of the same tube, we incubated 60 μL Lipofectamine™ CRISPAMAX™ reagent in 40 μL Opti-MEM media at RT for 1 minute. The Lipofectamine™ CRISPAMAX™ reagent was then added to the sgRNA/Opti-MEM™ I solution, mixed well with pipetting and allowed to incubate for a period of 15 minutes. This transfection complex was then added to the adherent A549 cells with 50 μL per well for the 24 well plate. For the chiral $(GHGXY)_4$ coacervates, similar concentrations of sgRNA and Cas9 Protein was made in 1× PBS, allowed to incubate for 15 minutes and then 50 μL added per well for each treatment. The cells were incubated for 3 days at 37°C. At the end of 3 days, the culture medium was removed, cells were washed with PBS and genomic cleavage detection assay was performed using Flow Cytometry analysis.

Flow cytometry

The transfected A549 cells were washed with FACS wash buffer (FWB; 1× PBS with 10% FBS) and then transferred into a round-bottom 96-well plate for staining. Cells were washed again with FWB and then resuspended in 100 µl of PBS with eBioscience™ Fixable Viability Dye eFluor™ 506 antibody. The staining was quenched with FWB following a 20 min incubation. The cells were centrifuged, washed 2× times with FWB, and resuspended in 100 µl FWB with CD55 (DAF) Antibody (anti-human; miltenyibiotec Cat#130-126-382). After 20 min, staining was quenched, cells washed twice and resuspended in 200 µl FWB for acquisition. Single-stained cells stained with eFluor™ 506, CD55 (DAF) Antibody, were used as compensation controls. Samples were acquired using an Agilent NovoCyte 3000 flow cytometer. Data were analyzed with FlowJo version 10. Proliferation was quantified as the percentage $CD55^+$ cells, considering untreated cells as the negative control and Lipofectamine treated ones as the positive control.

Antigen delivery to lysosomes and inhibitor effects

DC 2.4 cells were cultured in RPMI-1640 (Catalog number: 11875093) medium supplemented with 10% FBS, 100 U/mL penicillin, 100 µg/mL streptomycin, and 1 mM sodium pyruvate. Cells were plated in 8-well chamber slides (5000 cells in 200 µL of media) and incubated for 24 h prior to addition of DQ-OVA loaded coacervates for 2 h, 6 h, or 24 h (final DQ-OVA concentration was 2.5 µg/mL). Cells treated with PBS or soluble OVA served as controls. Cells were then washed and fixed using 4% Paraformaldehyde (PFA) for 15 min at RT and permeabilized with 0.1% Triton X-100 for 5 min prior to nuclear staining with Hoechst stain (Catalog number: 62249, Thermo Fischer Scientific; 10 µg/mL). Cells were imaged (60×) on Lionheart FX automated microscope (BioTek Instruments, Inc.) with controlled exposure

parameters across two channels: eGFP and DAPI. Images were captured for each time point (2, 6, and 24 h) to assess the cellular uptake and lysosomal processing of DQ-OVA. The images were deconvoluted using Gen 5 software overlaid to visualize localization. To test the effect of endocytic inhibitors on coacervate uptake, BMDCs (5×10^4 cells/mL in 96-well plates) were pre-treated with Chlorpromazine (30 µM), Dynasore (50 µM), Wortmannin (10 µM), IPA-3 (10 µM), or Methyl β-CD (2 mM) for 1 h prior to the addition of OVA-loaded coacervates. After 6 h, cells were washed and overlaid with DOBW cells for 16 h and IL-2 production was assessed by ELISA as described above. The effect of MHC-I inhibitors, Bortezomib (10 nM), Brefeldin A (17.8 µM) and Lactacystin (50 µM) or MHC-II inhibitors NH_4Cl (20 mM), Bafilomycin (500 nM), and 3-Methyl Adenine (10 µM) were tested similarly, using appropriate hybridomas for MHC-II (DOBW) or MHC-I (OVA 1.3) presentation.

T cell proliferation and functional cytokine production

Cultured mouse BMDCs (7-9 days) were plated in 96-well plates at a concentration of 5×10^4 cells per well and treated with OVA loaded coacervates for 24 h. Spleens and lymph nodes were harvested from OT-I/OT-II transgenic mice and single-cell suspensions were prepared by mechanical dissociation in EasySep Buffer (1× DPBS, 2% FBS, 1 mM EDTA). Cells were centrifuged at 300 × *g* for 10 min, counted using trypan blue exclusion staining, and resuspended at 1×10^8 cells/mL in EasySep Buffer. $CD8^+$/$CD4^+$T cells (for OT-I/OT-II mice) were isolated from this suspension using the EasySep Mouse $CD8^+$/$CD4^+$ T Cell Isolation kit (STEMCELL Technologies; Cat#19852 for $CD4^+$T; Cat#19853 for $CD8^+$T) *via* negative selection using the Big Easy EasySep magnet (STEMCELL Technologies; Cat#18001) following the manufacturer's instructions. Isolated $CD8^+$/$CD4^+$T cells were washed twice with 1× PBS, counted again, and

resuspended at 5×10^6 cells/mL in 1× PBS containing 2 µM Cell Trace Violet (CTV, Thermo Fisher, USA Cat#C34571). Cells were incubated at 37°C (protected from light) for 20 min, after which 5× staining volume of EasySep Buffer was added for another 5 min of incubation. The cells were counted again, centrifuged at 300 *g* for 5 min, resuspended in RPMI-1640 (Gibco 31800-022) with 10% FBS (R and D Systems S11150H), 10 mM HEPES (Sigma H0887), 1× MEM NEAA (Gibco 11140-050), 1 mM sodium pyruvate (Gibco 11360-070), 1× pen-strep glutamine (Gibco 10378-016), and 55 µM β-mercaptoethanol (Gibco 21985-023) added fresh, and then plated onto treated BMDCs. The plates were incubated for ~66 h, after which supernatants were collected for Mouse Adaptive Immune CodePlex Secretome analyses (Bruker Cellular Analysis, Inc), and cells were collected for flow cytometry studies.

Flow cytometry

Total cells from the spleen and lymph nodes of OT-I/OT-II mice were washed with FACS wash buffer (FWB; 1× PBS with 10% FBS) and transferred into a round-bottom 96-well plate for staining. Cells were washed again with 1×PBS and then resuspended in 100 µl of PBS with Zombie NIR (for OT-I; Biolegend Cat#423105) and 7-AAD (for OT-II; Biolegend Cat#420404) and anti-mouse CD16/32 (Biolegend Cat#101302) antibody. After a 30 min incubation, the staining was quenched. The cells were centrifuged, washed, and resuspended in 100 µl FWB with APC anti-mouse CD8 antibody (Biolegend Cat#17-0081-82) and APC/Cy7 (Biolegend Cat#100413) anti-mouse CD4 antibody for OT-I and OT-II mice, respectively. After 30 min, staining was quenched, cells washed twice, and resuspended in 200 µl FWB for acquisition. Single-stained cells or UltraComp eBeads (Invitrogen Cat#01-3333-42) stained with Zombie NIR, CTV, or CD8 and 7-AAD, CTV, or CD4 APC/Cy7 were used as compensation controls for OT-I and OT-II mice,

respectively. Samples were acquired using an Agilent NovoCyte 3000 flow cytometer. Data were analyzed with FlowJo version 10. Proliferation was quantified as the percentage of daughter cells out of single, live, $CD4^+$ or $CD8^+$T cells, gated as having a lower CTV fluorescence intensity than the peak of highest fluorescence (undivided).

Statistical analysis

Statistical analysis was performed in GraphPad Prism. Data are expressed as mean ± S.E.M. and statistical analysis was performed using a one-way or two-way ANOVA with Tukey/Sidak's/Dunnett's multiple comparison test. *$p \leq 0.05$, **$p \leq 0.01$, ***$p \leq 0.001$, ****$p \leq 0.0001$.

Peptides	Sequence	LLPS	AHI
$(GHGXY)_4$ X=F, GF 20	GHG**F**YGHG**F**YGHG**F**YGHG**F**Y	No	-1.06
$(GHGXY)_4$ X=L, GL 20	GHG**L**YGHG**L**YGHG**L**YGHG**L**Y	Yes	-0.92
$(GHGXY)_4$ X=I, GI 20	GHG**I**YGHG**I**YGHG**I**YGHG**I**Y	Yes	-0.92
$(GHGXY)_4$ X=V, GV 20	GHG**V**YGHG**V**YGHG**V**YGHG**V**Y	Yes	-0.86
$(GHGXY)_4$ X=A, GA 20	GHG**A**YGHG**A**YGHG**A**YGHG**A**Y	No	-0.66
$(GHGXY)_4$ X=P, GP 20	GHG**P**YGHG**P**YGHG**P**YGHG**P**Y	Yes	-0.56
$(GHGXY)_4$ X=N, GN 20	GHG**N**YGHG**N**YGHG**N**YGHG**N**Y	No	-0.52

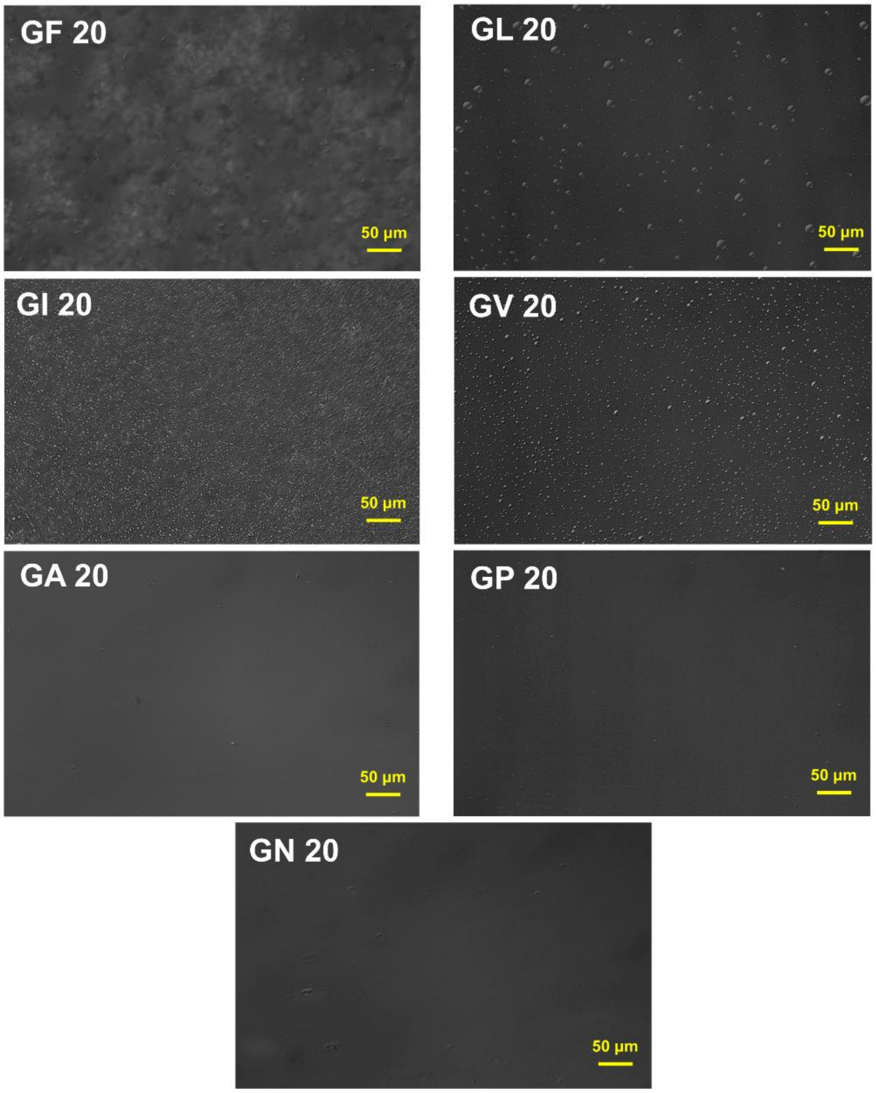

Figure S1: Peptide sequences used in this study along with their average hydrophilicity index (AHI) and their optical microscopy images to depict droplet formation.

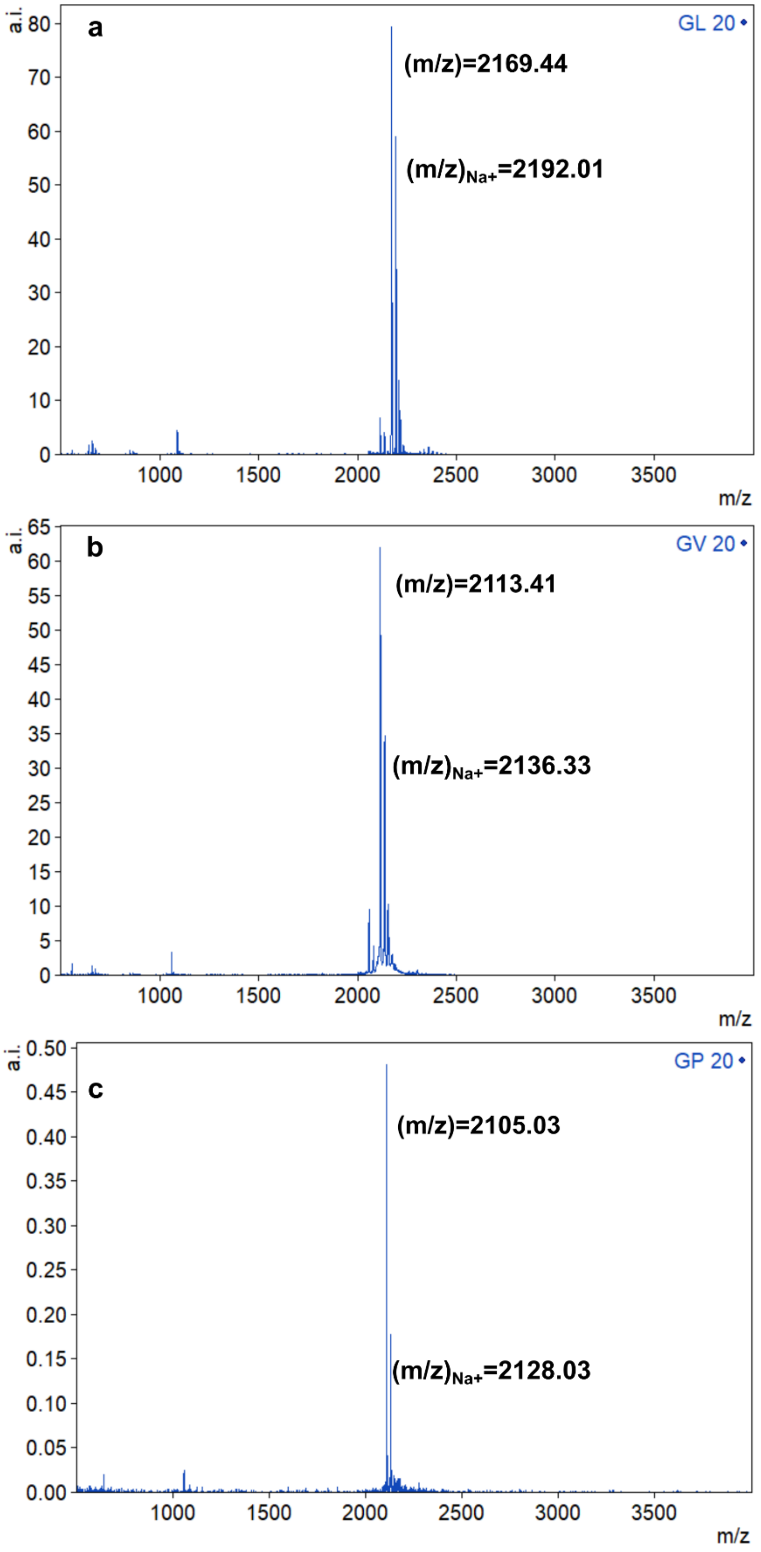

Figure S2: MALDI-TOF spectra of L- (a) $(GHGLY)_4$, (b) $(GHGVY)_4$, and (c) $(GHGPY)_4$.

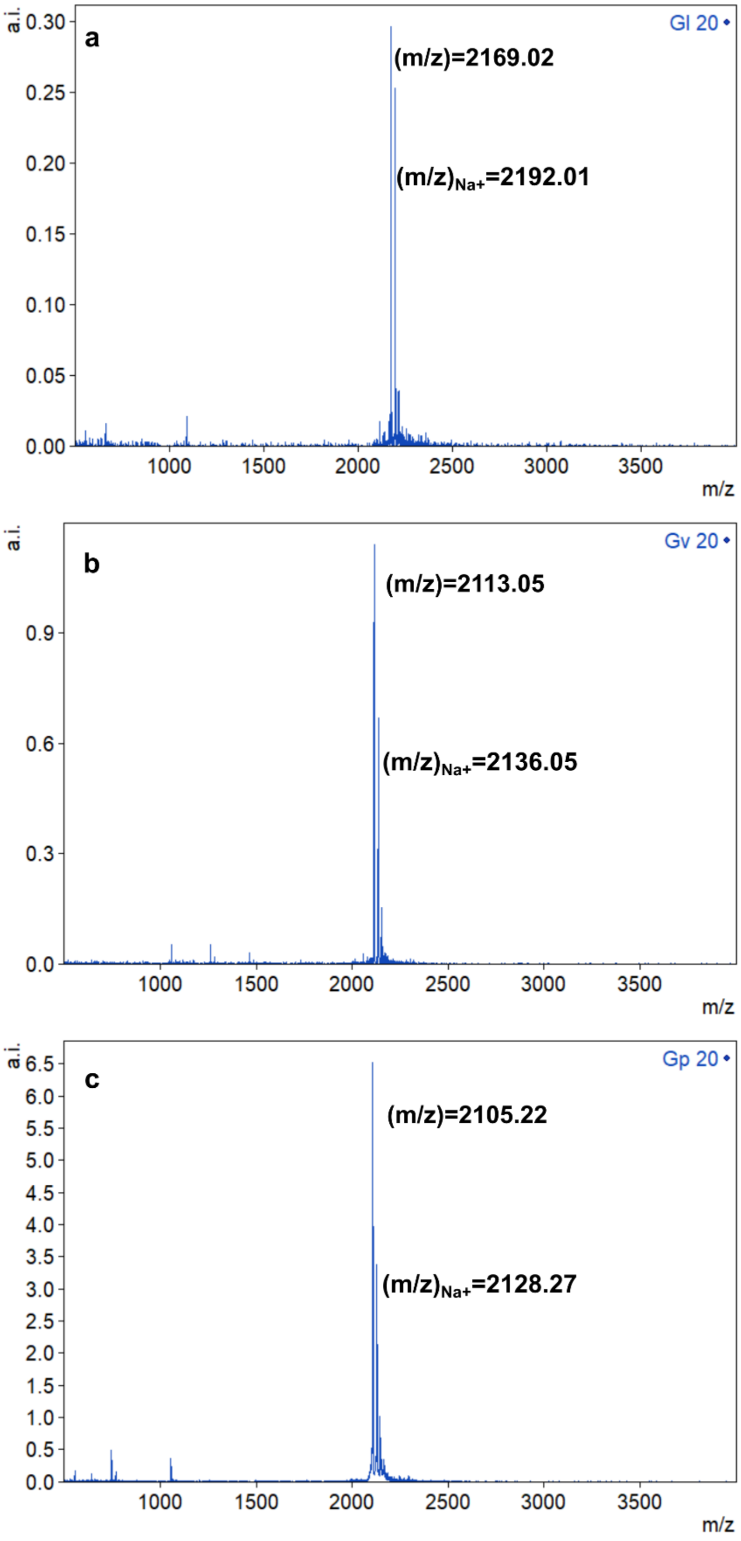

Figure S3: MALDI-TOF spectra of D- (a) $(GHGLY)_4$, (b) $(GHGVY)_4$, and (c) $(GHGPY)_4$.

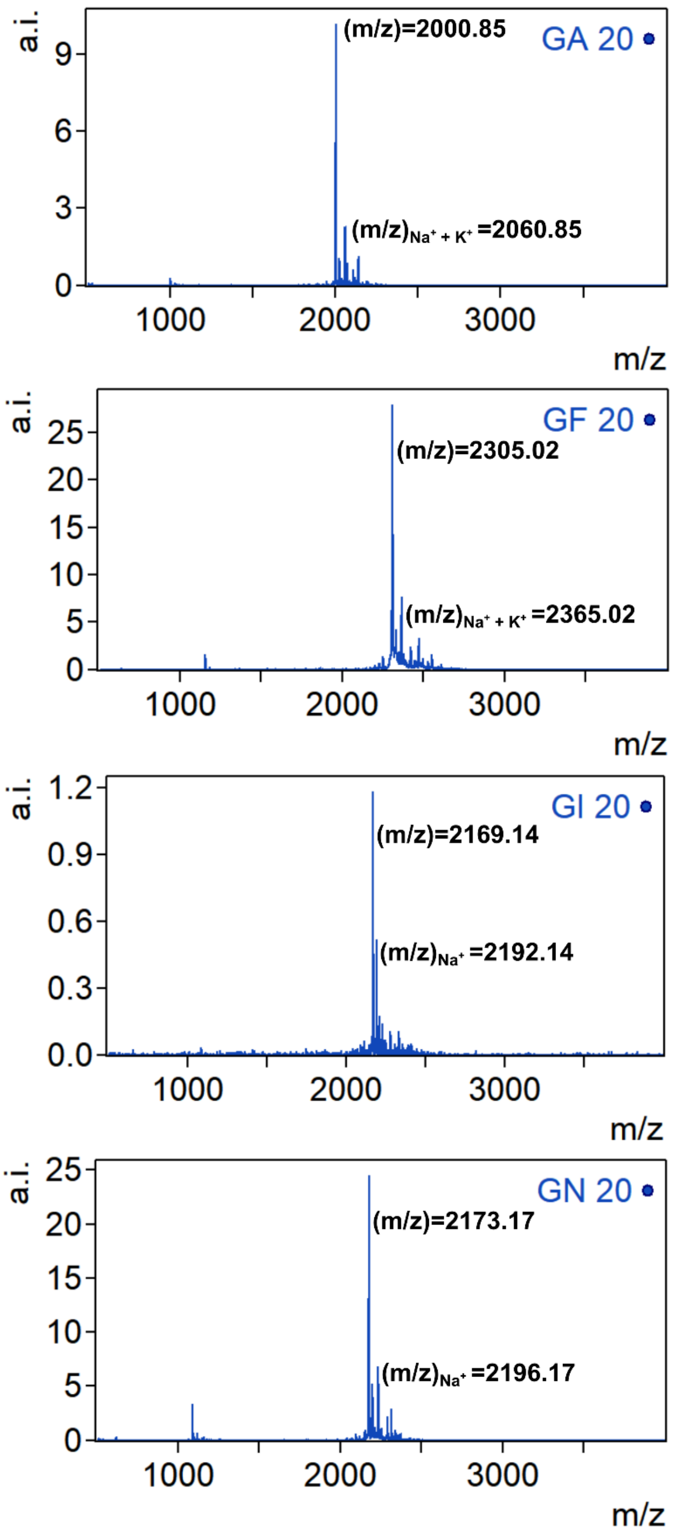

Figure S4: MALDI-TOF spectra of $(GHGAY)_4$, $(GHGFY)_4$, $(GHGIY)_4$, and $(GHGNY)_4$.

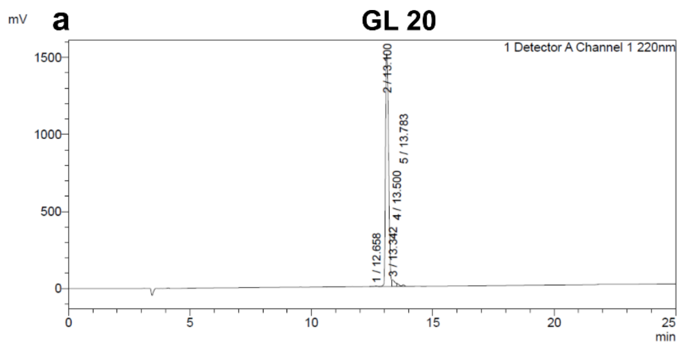

<Peak Table>

Detector A Channel 1 220nm

Peak#	Ret. Time	Area	Height	Area%
1	12.658	39628	3777	0.282
2	13.100	13475481	1511158	95.787
3	13.342	351821	42073	2.501
4	13.500	136710	20290	0.972
5	13.783	64580	10208	0.459
Total		14068219	1587506	100.000

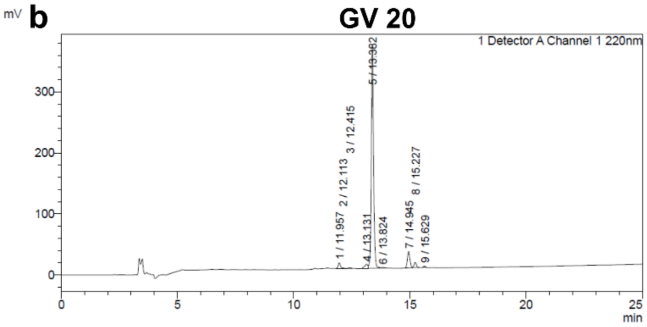

<Peak Table>

Detector A Channel 1 220nm

Peak#	Ret. Time	Area	Height	Area%
1	11.957	64244	9410	2.237
2	12.113	3791	740	0.132
3	12.415	5415	782	0.189
4	13.131	63347	6862	2.206
5	13.382	2445530	362637	85.156
6	13.824	10971	1103	0.382
7	14.945	204369	26810	7.116
8	15.227	61153	8712	2.129
9	15.629	13017	2386	0.453
Total		2871837	419441	100.000

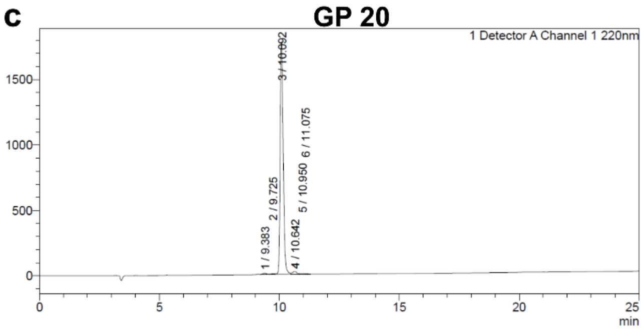

<Peak Table>

Detector A Channel 1 220nm

Peak#	Ret. Time	Area	Height	Area%
1	9.383	106871	11247	0.657
2	9.725	36779	4053	0.226
3	10.092	15754338	1777601	96.806
4	10.642	270474	21080	1.662
5	10.950	75355	5433	0.463
6	11.075	30393	4210	0.187
Total		16274210	1823623	100.000

Figure S5. HPLC chromatograms of purified L- (a) $(GHGLY)_4$, (b) $(GHGVY)_4$, and (c) $(GHGPY)_4$.

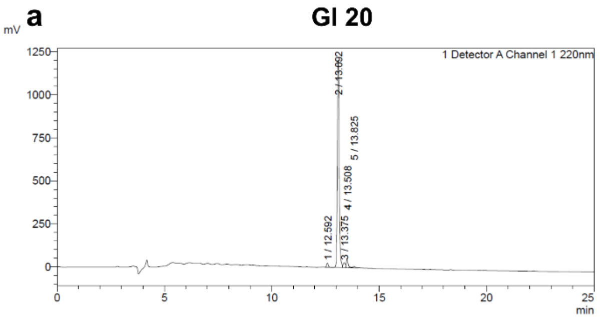

<Peak Table>

Detector A Channel 1 220nm

Peak#	Ret. Time	Area	Height	Area%
1	12.592	134077	24905	1.641
2	13.092	7451169	1205003	91.209
3	13.375	186507	28836	2.283
4	13.508	318592	46521	3.900
5	13.825	78960	7615	0.967
Total		8169304	1312879	100.000

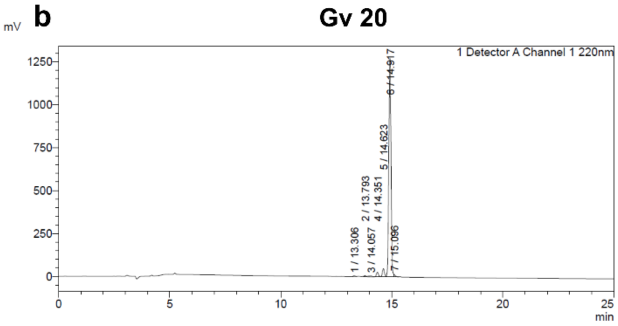

<Peak Table>

Detector A Channel 1 220nm

Peak#	Ret. Time	Area	Height	Area%
1	13.306	23680	4871	0.248
2	13.793	38065	6583	0.398
3	14.057	55123	5978	0.576
4	14.351	182552	26888	1.908
5	14.623	320663	47801	3.352
6	14.917	8885953	1273403	92.885
7	15.096	60625	16105	0.634
Total		9566662	1381629	100.000

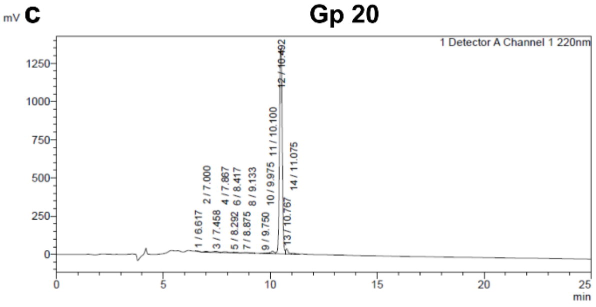

<Peak Table>

Detector A Channel 1 220nm

Peak#	Ret. Time	Area	Height	Area%
1	6.617	25305	2812	0.188
2	7.000	67005	4907	0.499
3	7.458	103599	5944	0.771
4	7.867	90073	5372	0.671
5	8.292	17985	2755	0.134
6	8.417	48342	3768	0.360
7	8.875	70849	4092	0.527
8	9.133	31129	2787	0.232
9	9.750	44469	3293	0.331
10	9.975	31311	6288	0.233
11	10.100	177567	17195	1.322
12	10.492	12365770	1345508	92.063
13	10.767	282163	33950	2.101
14	11.075	76228	5766	0.568
Total		13431794	1444436	100.000

Figure S6. HPLC chromatograms of purified D- (a) $(GHGLY)_4$, (b) $(GHGVY)_4$, and (c) $(GHGPY)_4$.

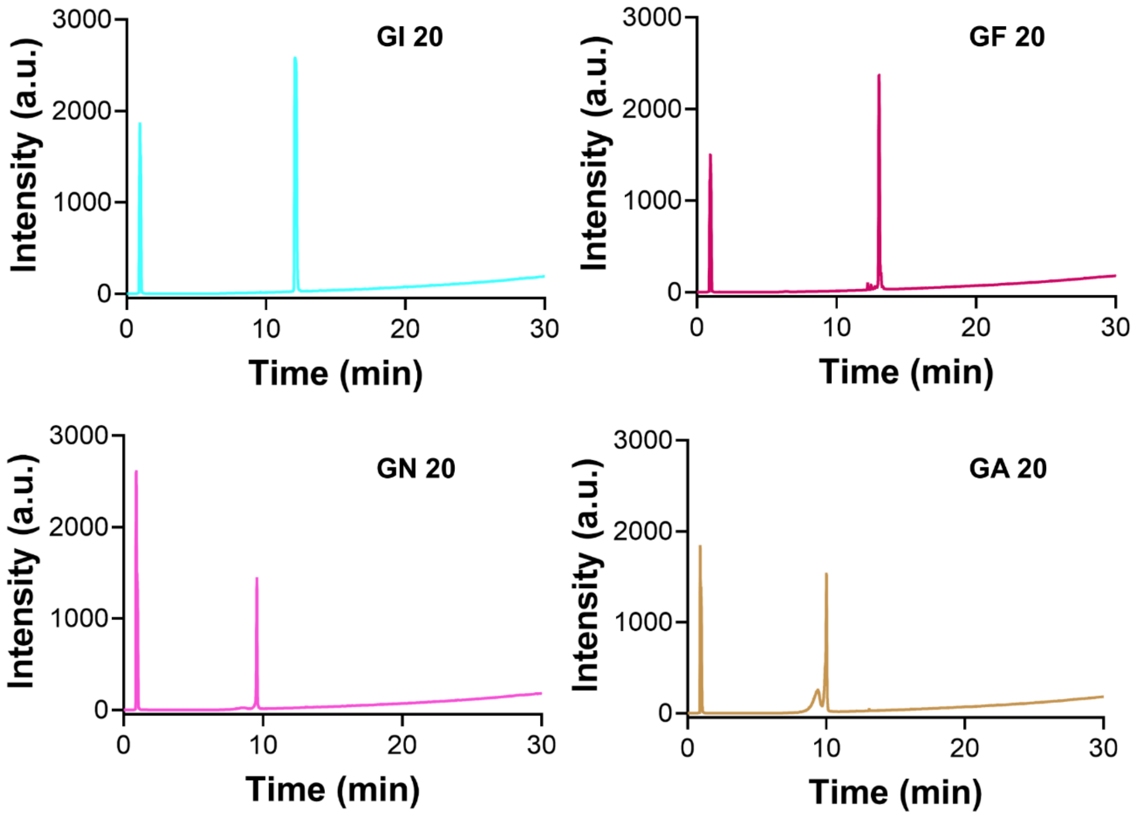

Figure S7. HPLC chromatograms of purified $(GHGIY)_4$, $(GHGFY)_4$, $(GHGNY)_4$, and $(GHGAY)_4$. Purity of $(GHGIY)_4$, $(GHGFY)_4$, $(GHGNY)_4$, and $(GHGAY)_4$ were 98%, 87%, 92% and 85% respectively. The peaks in all the profiles ~1 min represents solvent peak (50/50 v/v ACN/Water)

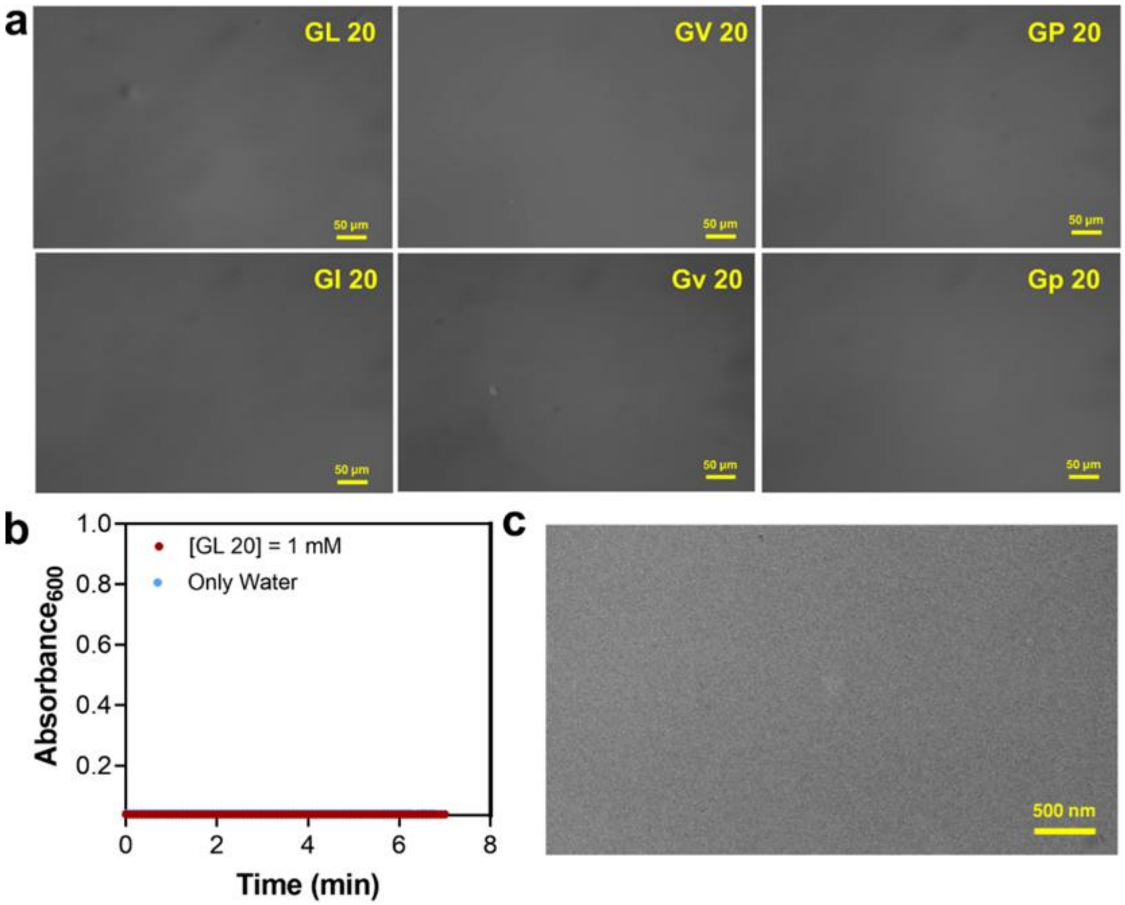

Figure S8. Optical images of (a) $(GHGLY)_4$, $(GHGVY)_4$, and $(GHGPY)_4$ and their enantiomers, (b) Kinetics of LLPS by 1 mM $(GHGLY)_4$ in water, and (c) TEM image of 1 mM $(GHGLY)_4$ in water.

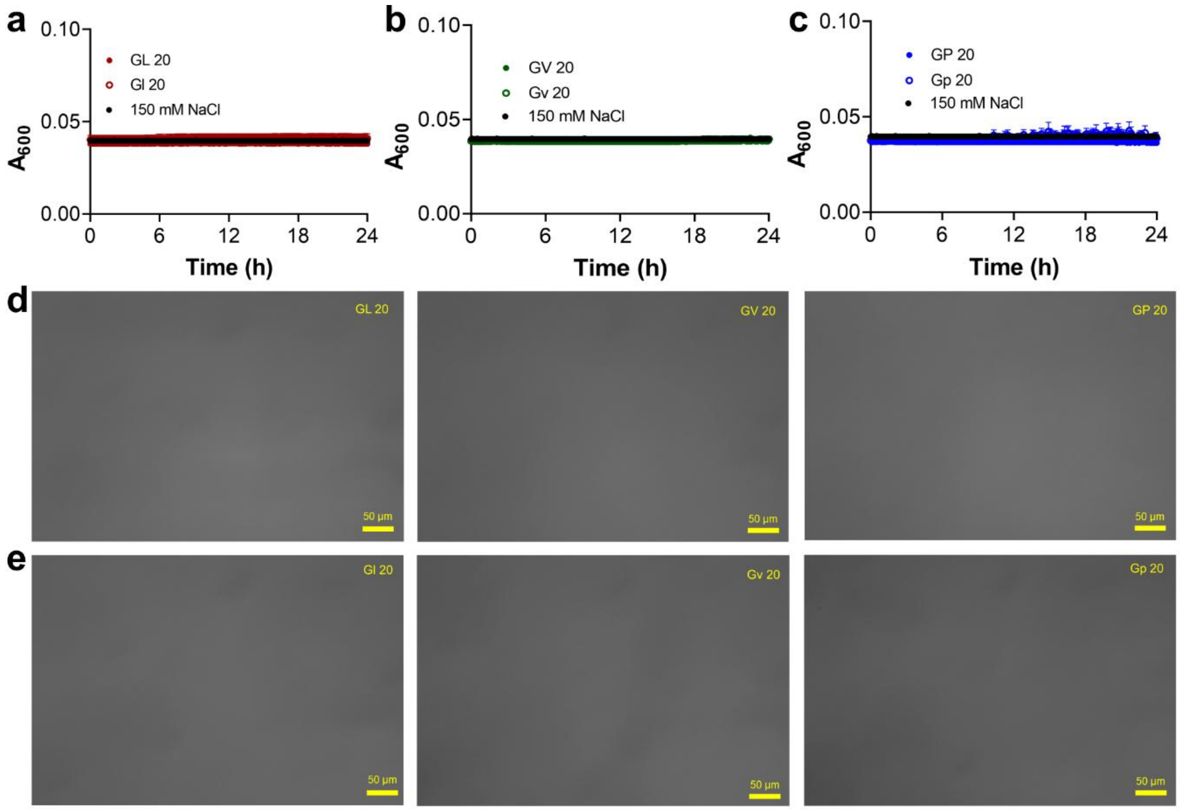

Figure S9. Turbidity measurements of (a) $(GHGLY)_4$, (b) $(GHGVY)_4$, (c) $(GHGPY)_4$ peptides and their enantiomers (1 mM, in 150 mM NaCl) and corresponding optical micrographs of (d) L- peptides and (e) D- peptides.

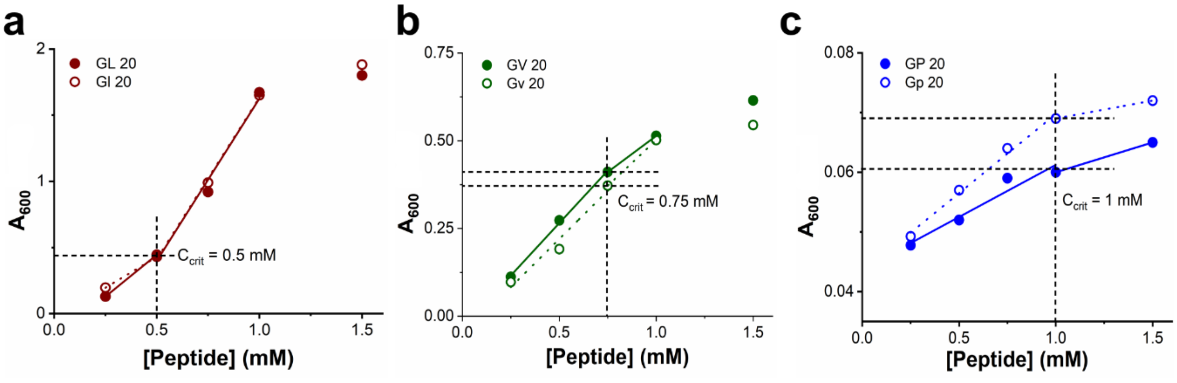

Figure S10. Determination of C_{crit} for (a) $(GHGLY)_4$, (b) $(GHGVY)_4$, (c) $(GHGPY)_4$ and (d-f) their corresponding enantiomers (1 mM, 1× PBS, pH 7.4).

Table S1. Measured C_{crit} values for $(GHGXY)_4$ peptides used in this study.

Peptide	C_{crit} (mM)
GL 20/Gl 20	0.5 mM
GV 20/Gv 20	0.75 mM
GP 20/Gp 20	1 mM

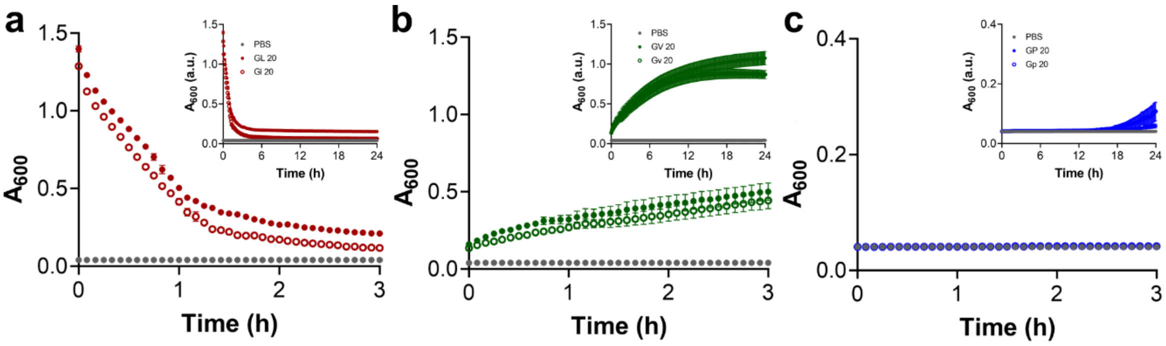

Figure S11. Turbidity measurements at 600 nm for (a) $(GHGLY)_4$, (b) $(GHGVY)_4$, (c) $(GHGPY)_4$ and their corresponding enantiomers (1 mM, 1×PBS, pH 7.4) without shaking. Inset shows measurements up to 24 h.

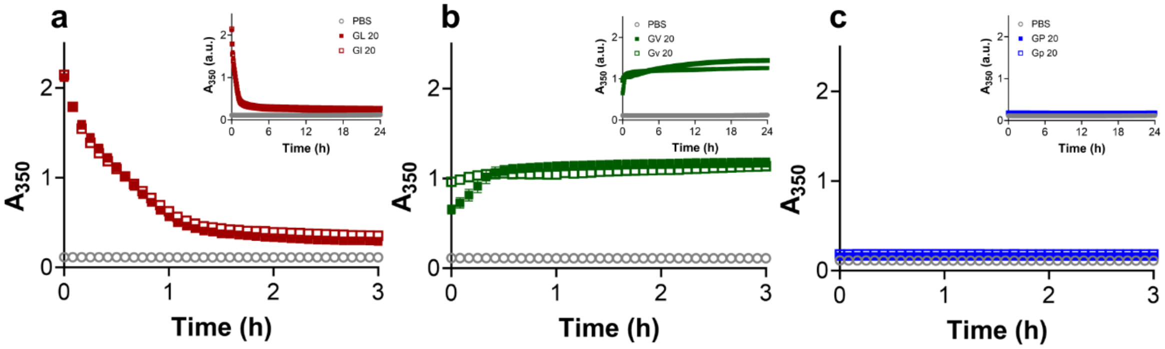

Figure S12. Turbidity measurements at 350 nm for (a) $(GHGLY)_4$, (b) $(GHGVY)_4$, (c) $(GHGPY)_4$ and their corresponding enantiomers (1 mM, 1× PBS, pH 7.4). Inset shows measurements up to 24 h.

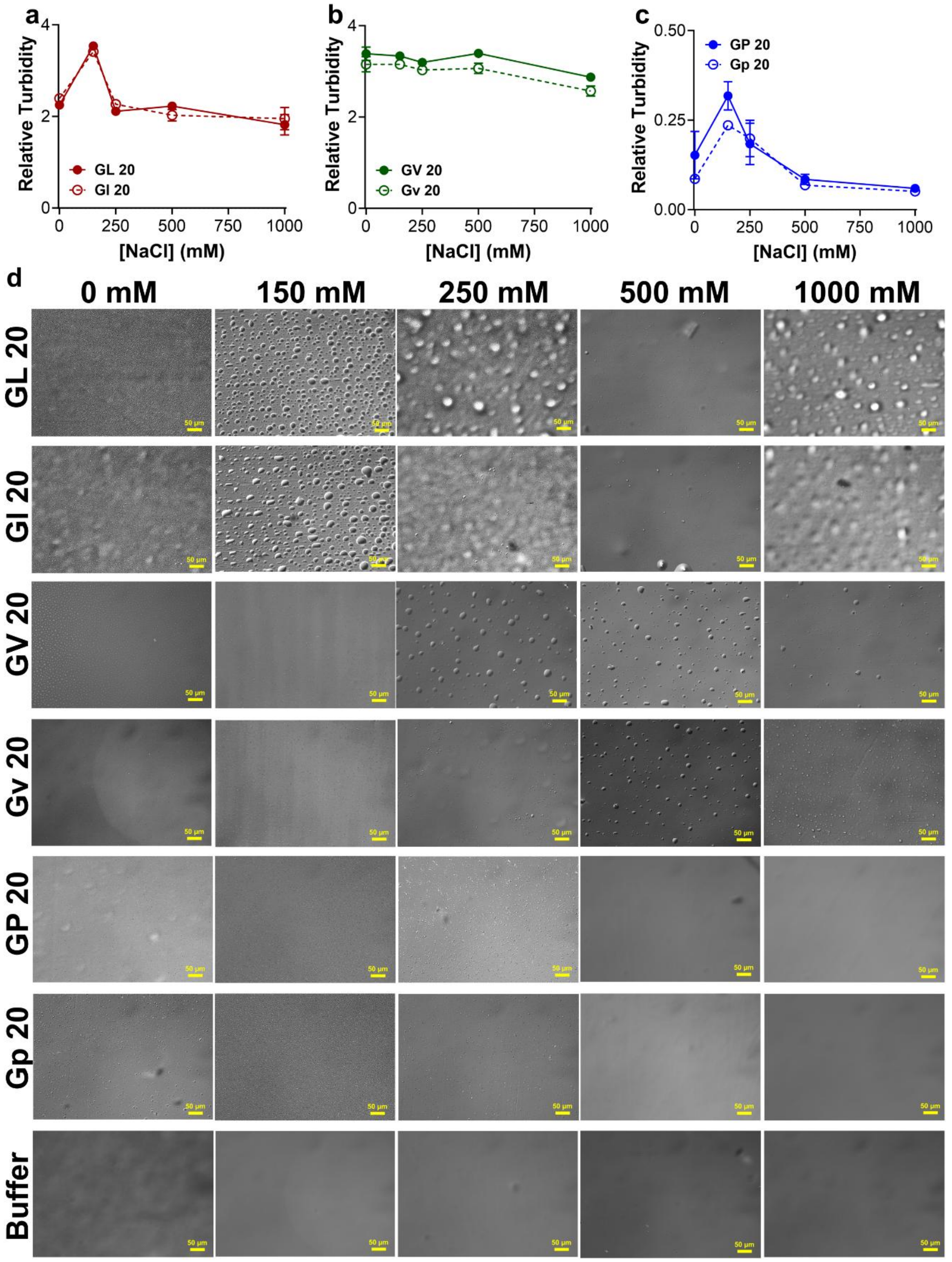

Figure S13. Relative turbidity (λ = 600 nm) of L- and D- peptides according to varied salt concentrations for (a) GL 20, (b) GV 20, and (c) GP 20. (d) Optical images of droplets formed by L- and D- $(GHGXY)_4$ variants at different salt concentrations (0-1000 mM) in 0.1 M phosphate buffer (pH 7.4).

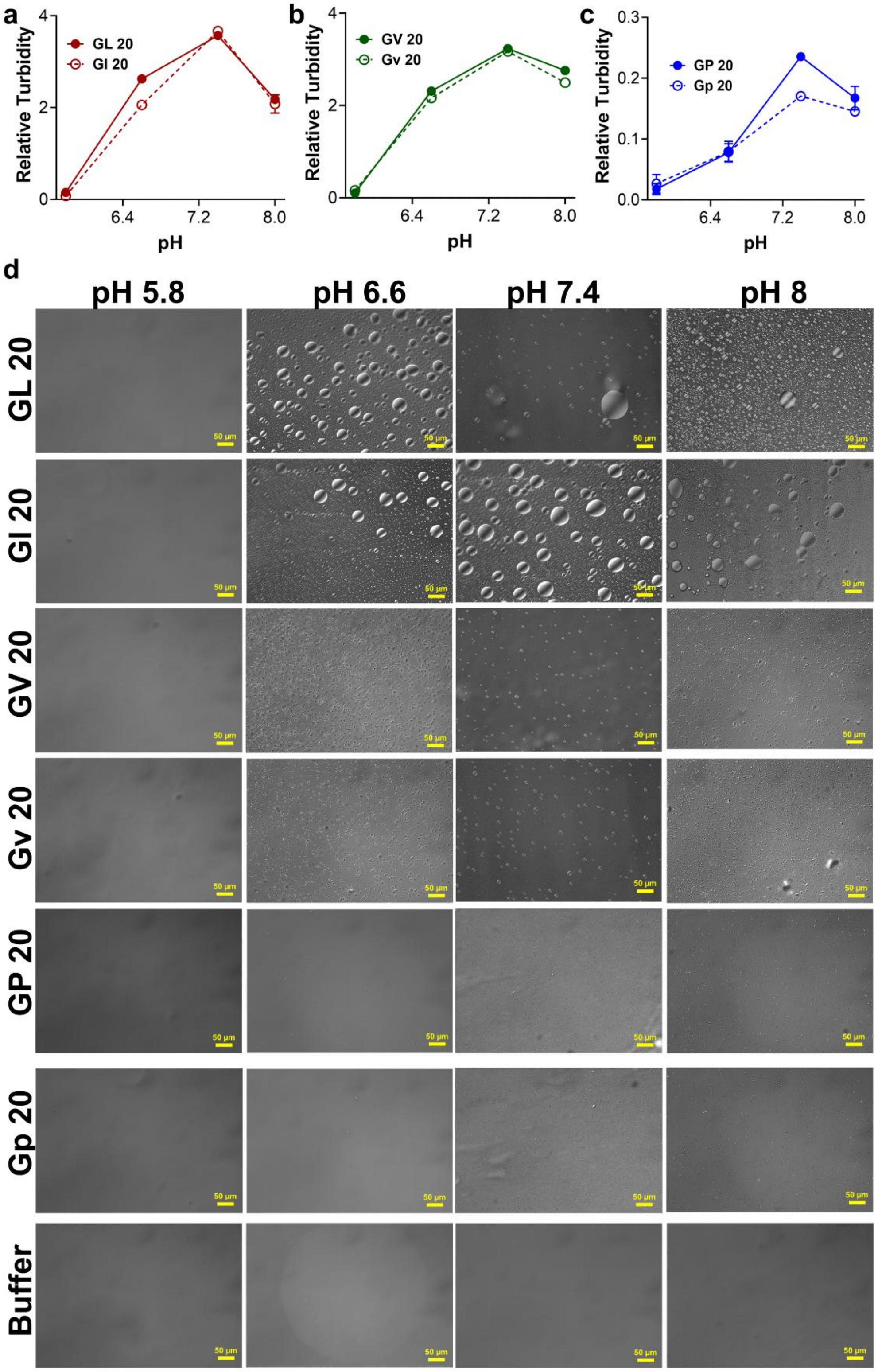

Figure S14. Turbidity measurements (λ = 600 nm) of L- and D- peptides at various pHs at 150 mM NaCl for (a) GL 20, (b) GV 20, and (c) GP 20. (d) Optical images of droplets formed by L- and D- of $(GHGXY)_4$ variants with varying pH of 0.1 M phosphate buffer.

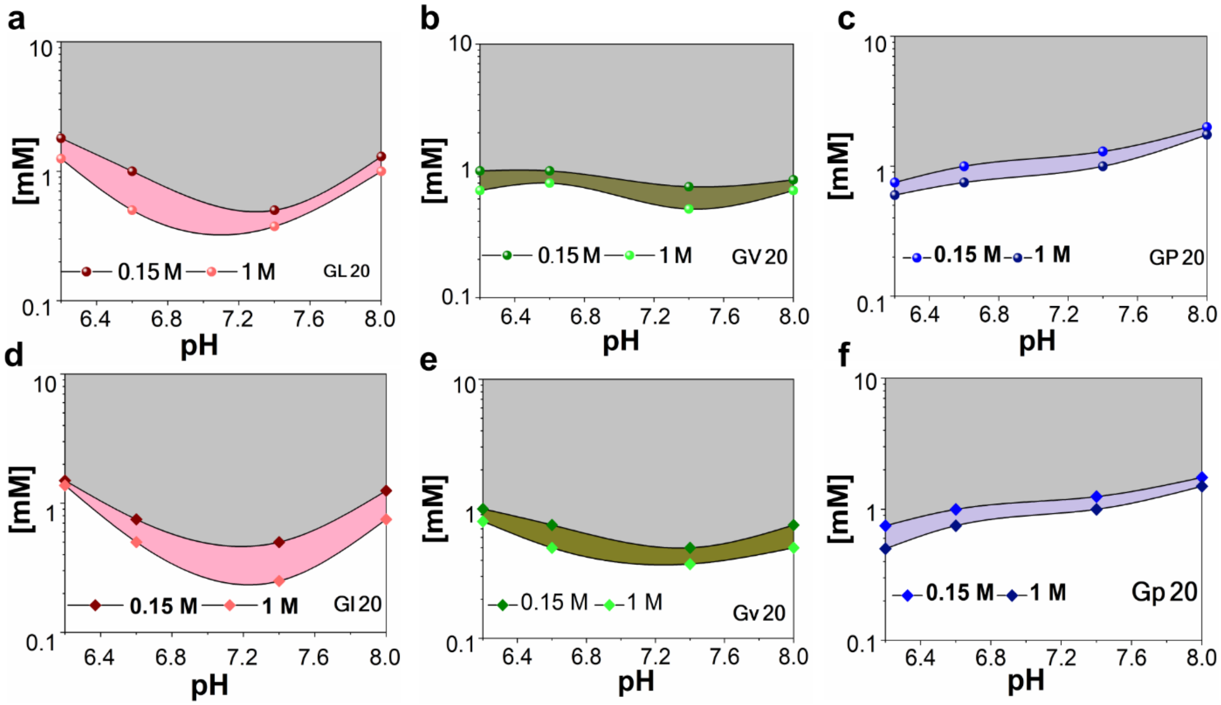

Figure S15. Critical phase separation concentrations of $(GHGXY)_4$ variants *vs.* pH at 0.15 M and 1 M NaCl for (a) GL 20, (b) GV 20, (c) GP 20 (d) Gl 20, (e) Gv 20, and (f) Gp 20.

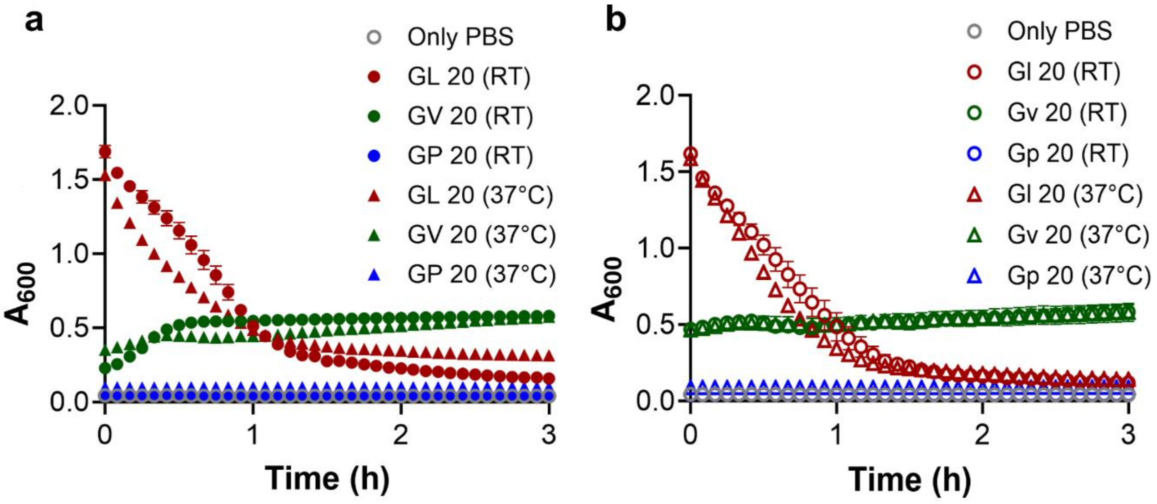

Figure S16. Turbidity measurements of (a) L- $(GHGXY)_4$ variants and (b) D- $(GHGXY)_4$ variants at 37°C (circles) and RT (triangles) in 1×PBS, pH 7.4.

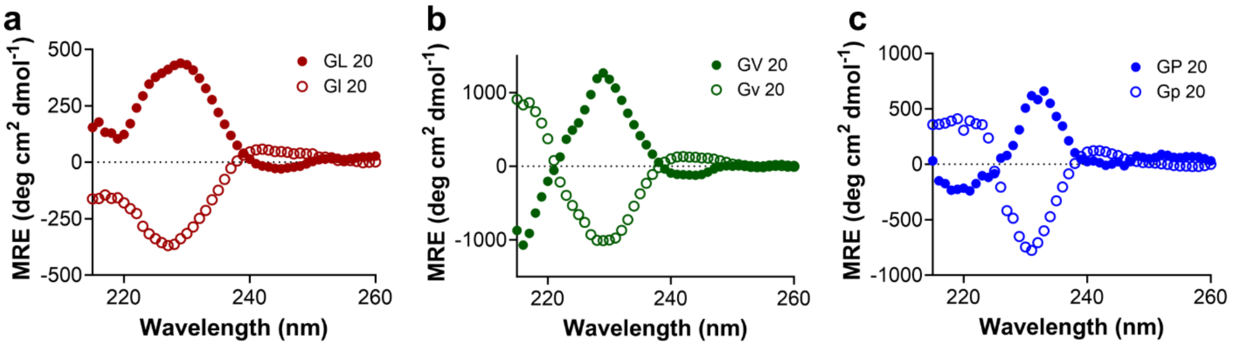

Figure S17. CD profiles of 0.75 mM L- and D- peptides- (a) GL 20 (b) GV 20, and (c) GP 20 in 1×PBS.

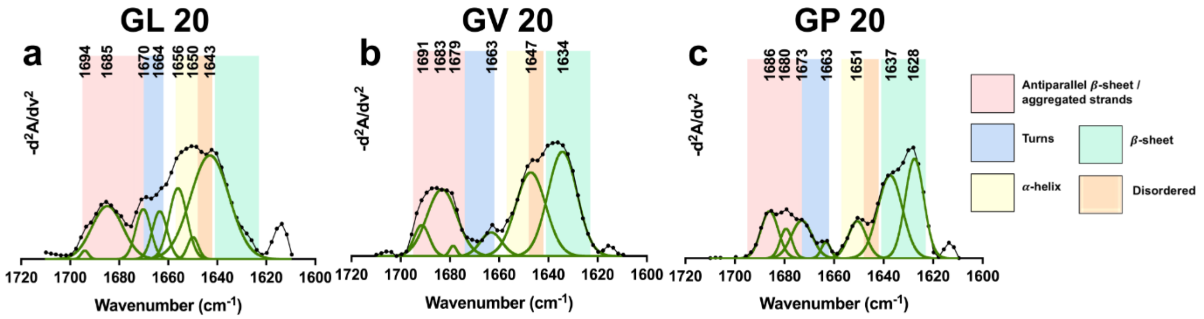

Figure S18. Second-derivative FT-IR spectra of (a) $(GHGLY)_4$, (b) $(GHGVY)_4$, and (c) $(GHGPY)_4$ peptide coacervates in 1×PBS (pH 7.4).

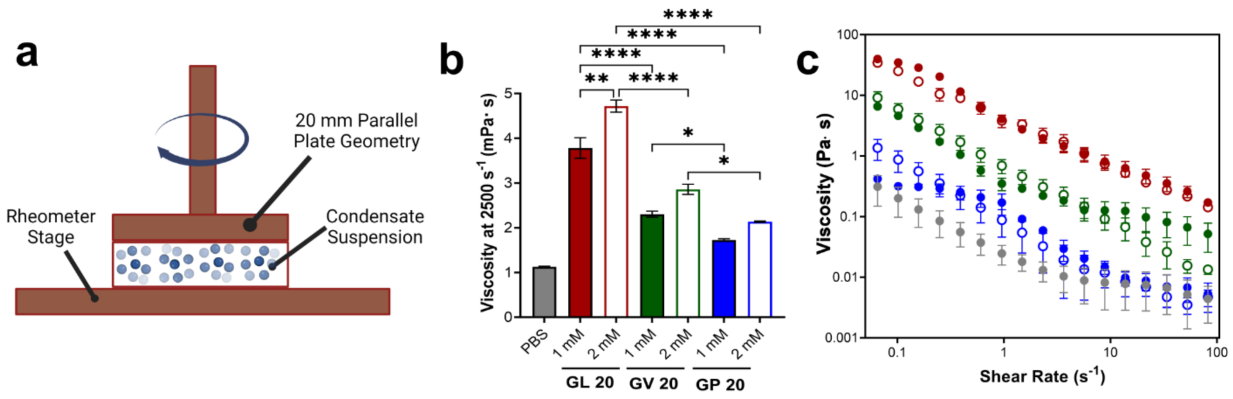

Figure S19. Mechanical testing of peptide coacervates. (a) Schematic of coacervate testing using a rotational rheometer. (b) Viscosity measured at a shear rate of 2500 s^{-1} using a Hagen-Poiseuille viscometer. Data is representative of three biological replicates and plotted as mean±SEM.

(c) Measured viscosity at varying shear rates using a rotational rheometer at a peptide coacervate concentration of 1 mM. The filled and open circles represent L- and D-GL 20 (red), GV 20 (green), GP 20 (blue), respectively. Grey circles represent 1×PBS alone.

*$p < 0.05$, **$p < 0.01$, ****$p < 0.0001$ as determined by a one-way ANOVA

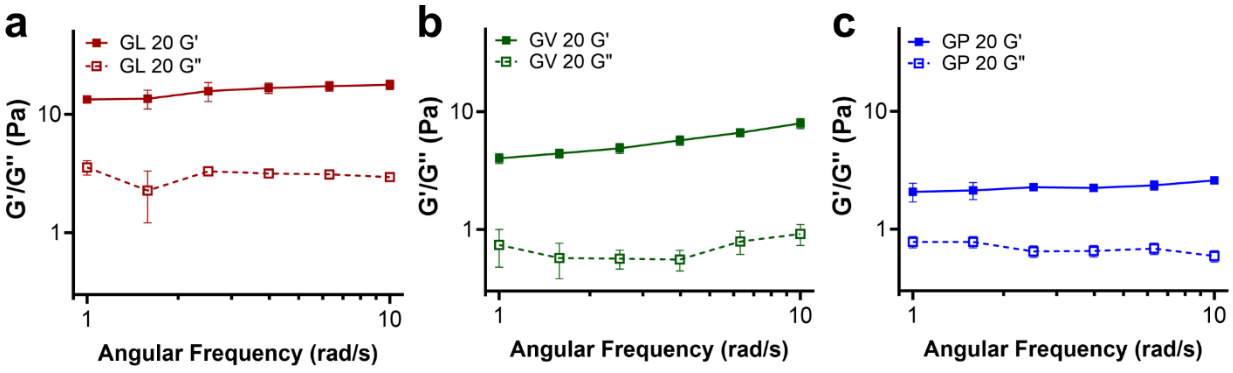

Figure S20. Storage (G') and loss modulus (G'') of (a) $(GHGLY)_4$, (b) $(GHGVY)_4$, and (c) $(GHGPY)_4$ coacervates (1 mM, 1×PBS, pH 7.4).

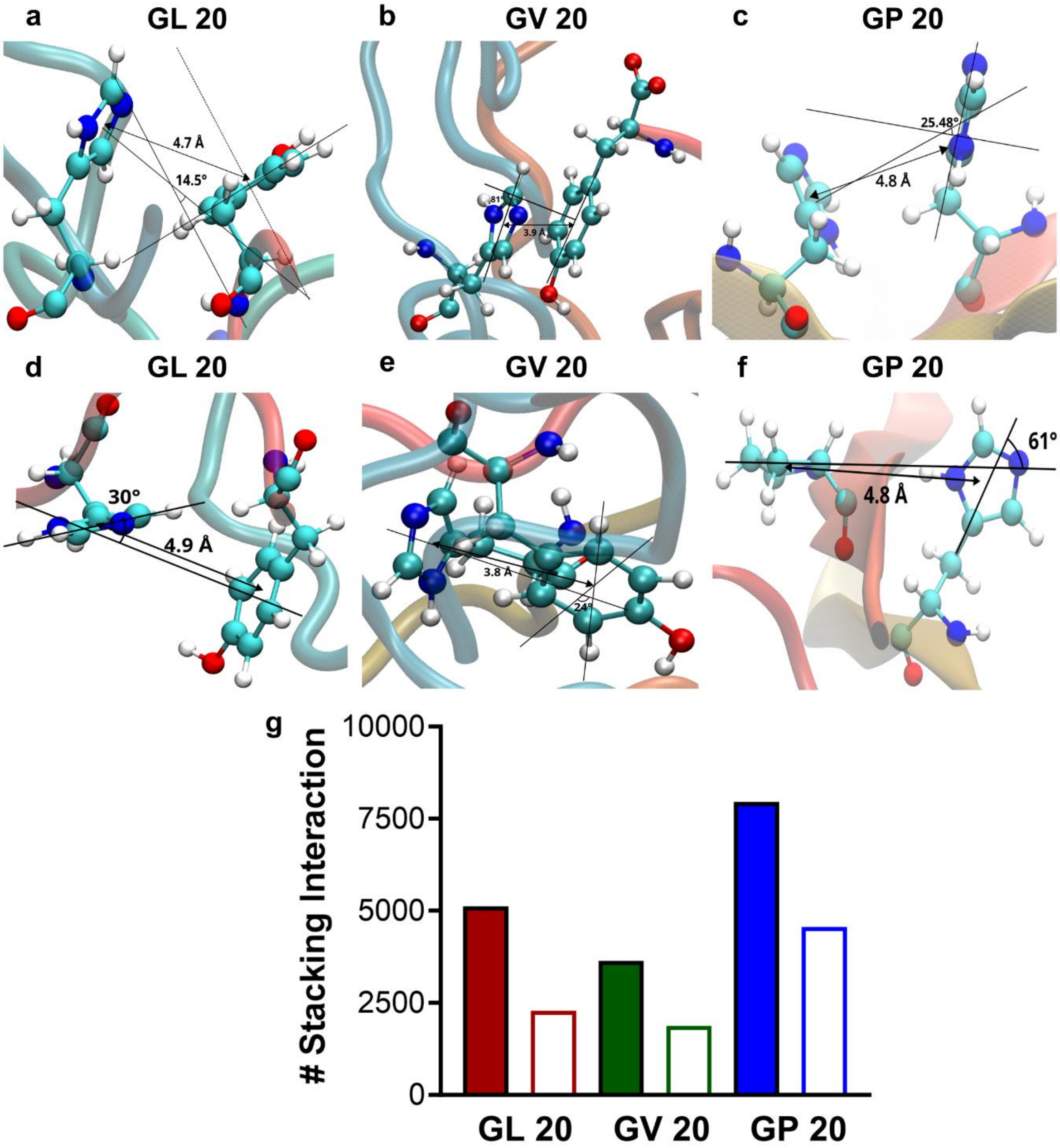

Figure S21. Parallel π-stacking interactions for (a) $(GHGLY)_4$, (b) $(GHGVY)_4$, and (c) $(GHGPY)_4$ peptides. Perpendicular π-stacking interactions for (d) $(GHGLY)_4$, (e) $(GHGVY)_4$, and (f) $(GHGPY)_4$ peptides. (g) Number of stacking parallel (solid bars) and perpendicular (clear bars) interactions occurred during the last 500 ns of simulation trajectories for all three peptides.

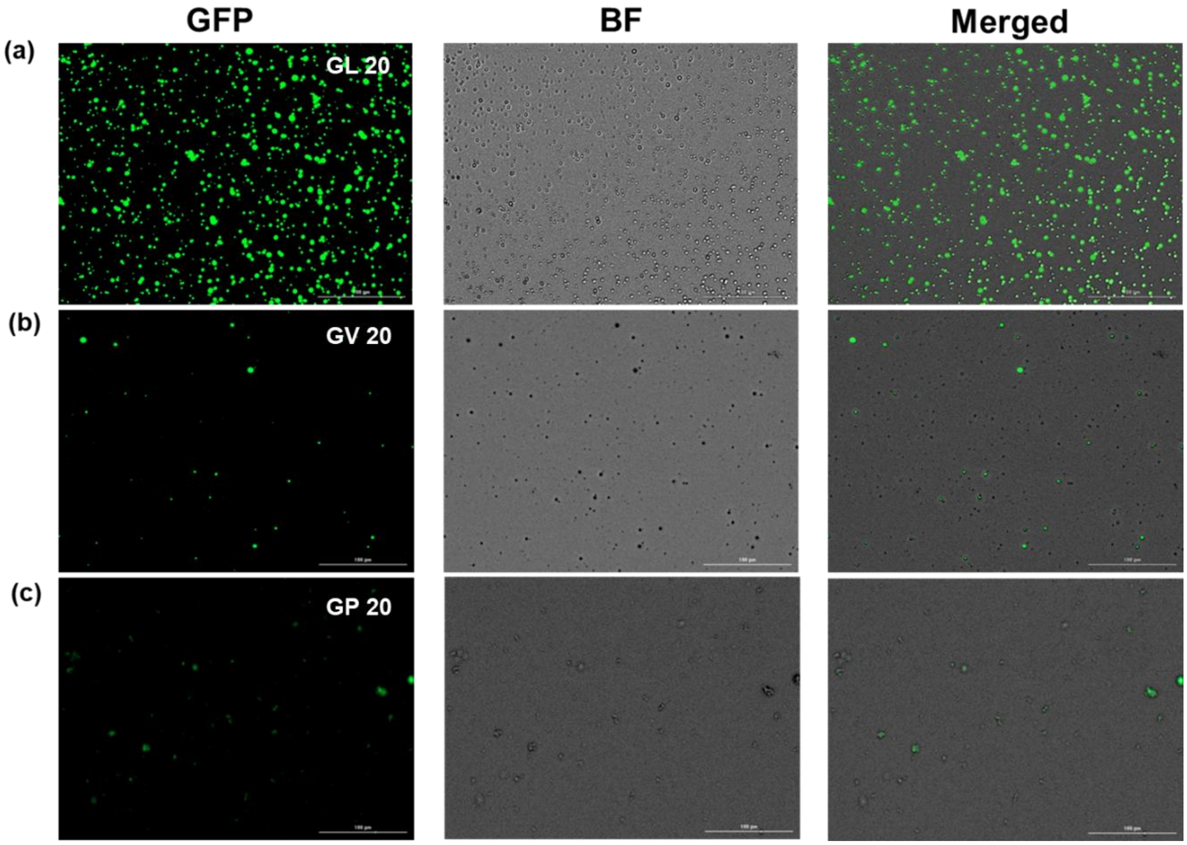

Figure S22. Fluorescence, bright field, and merged microscopy images of eGFP loaded (a) $(GHGLY)_4$, (b) $(GHGVY)_4$, and (c) $(GHGPY)_4$ coacervates. The left to right panels represent GFP, DIC and merged channels. Scale bar = 100 µm.

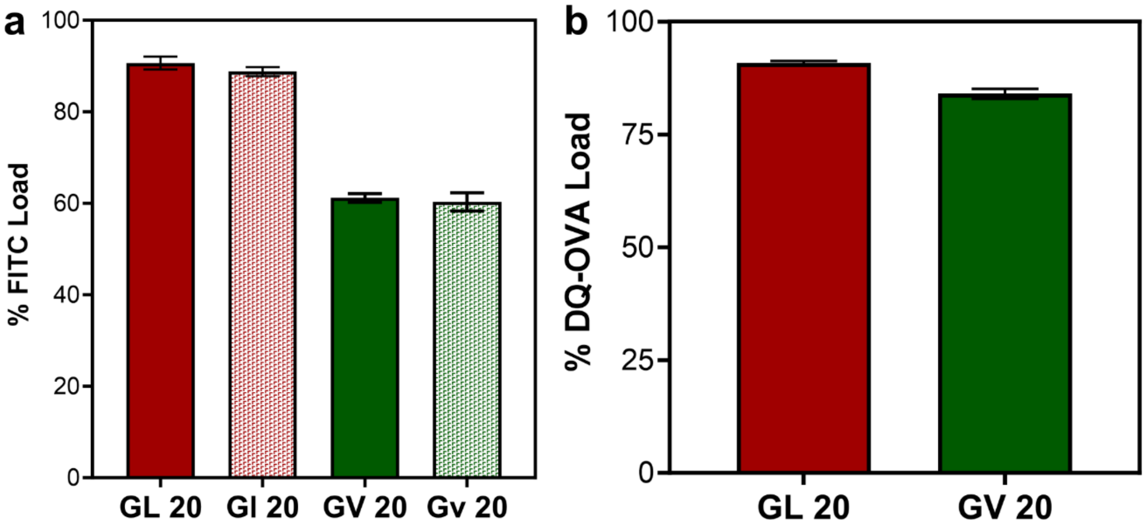

Figure S23. Encapsulation efficiency of (a) FITC-H-$2K^b$ antibody by leucine or valine coacervates and their enantiomers and (b) model antigen DQ-OVA load by leucine and valine droplets.

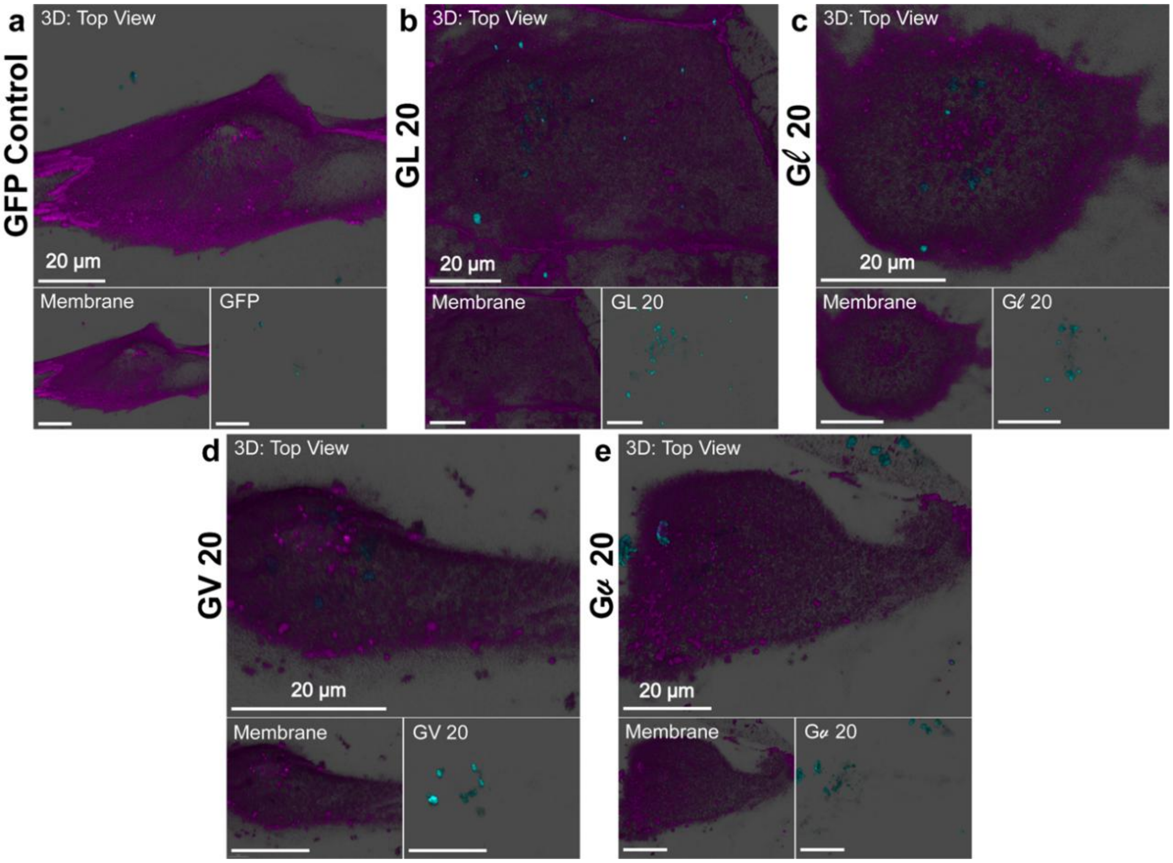

Figure S24. 3-D Top view of eGFP loaded coacervates in hiPSC-CM cultures. (a) GFP Control, (b, c) $(GHGLY)_4$ and its enantiomer, (d, e) $(GHGVY)_4$ and its enantiomer.

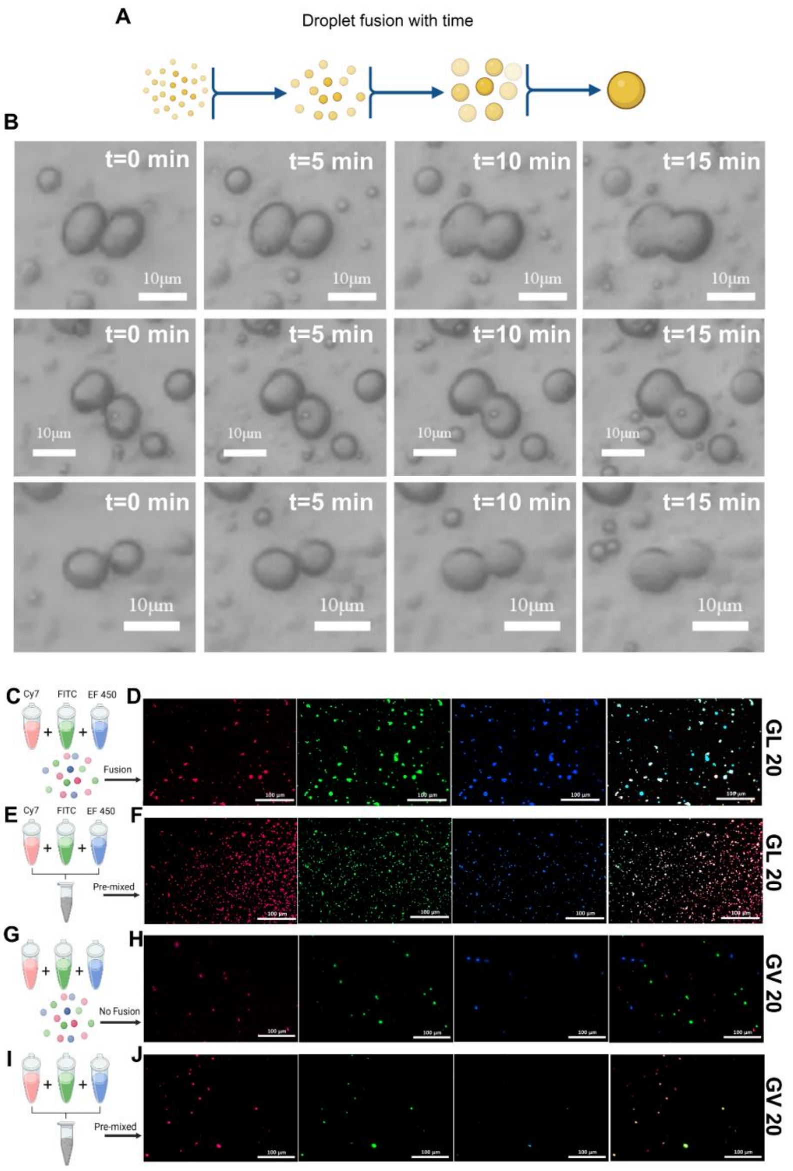

Figure S25. Droplet coalescence over time. (A) Schematic depicting the droplet coalescence over time to generate larger droplets. (B) The sequence of enhanced snapshots extracted from a continuous video recording captured the dynamic coalescence events of peptides GL 20 over 15 minutes. Each snapshot, chosen from frames taken every 500 milliseconds, highlights the coalescence process. The snapshots are time-stamped to document the progression and timing of each significant event, providing a detailed visual narrative of peptide behavior under experimental conditions. Schematic of fluorescently labeled cargo loaded in (C) GL 20 and (G) GV 20 coacervates and their subsequent coalescence over time. (D) Overlaid channels illustrate GL 20 coalescence over time, resulting in droplets of secondary color. The addition of premixed protein solutions to (E) GL 20 peptide and (I) GV 20 peptide is shown. (F) Overlaid channels show that GL 20 droplets encapsulate multiple proteins, as evidenced by secondary color and grey droplets. (H) Overlaid channels show that GV 20 droplets loaded with different fluorescent proteins do not coalesce over time and remain as single-colored droplets. (J) Overlaid channels indicate that GV 20 droplets cannot encapsulate multiple proteins when premixed.

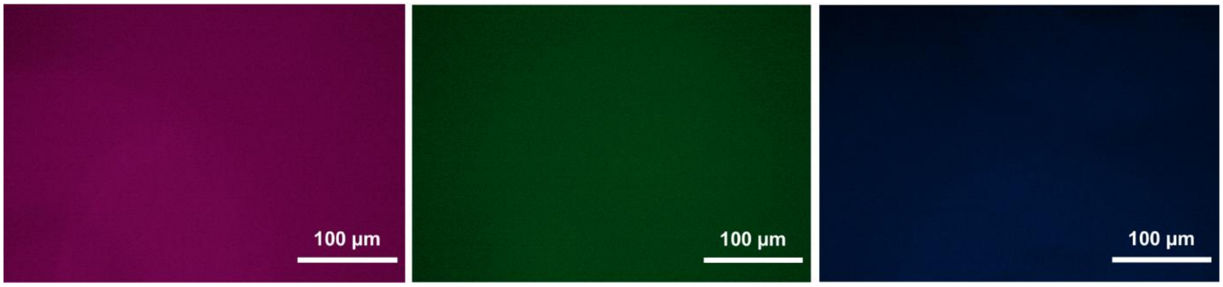

Figure S26. Microscopy images showing no fluorescent puncta without coacervates (Left-Red, Middle-Green, Right-Blue).

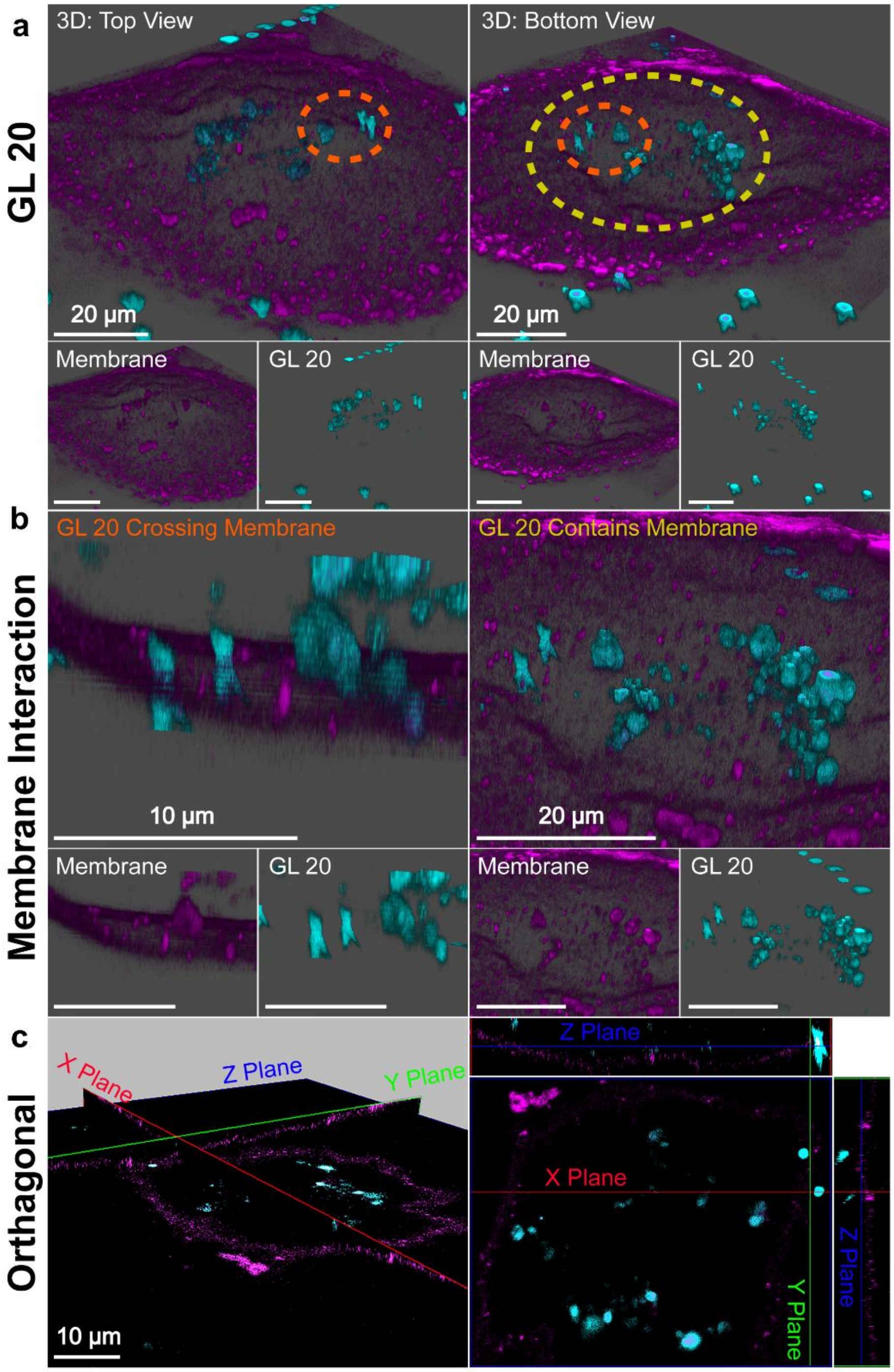

Figure S27. Membrane interactions of GL 20 in hiPSC-CMs. Representative confocal micrographs of hiPSC-CMs treated with GL 20 (teal). The cellular membrane was identified with WGA membrane stain (purple). (a) Micrographs are presented as 3D snapshots from the top and bottom views (scale: 20 µm). (b) Membrane interactions of GL 20 were captured as transmembrane crossings (orange, scale: 10 µm) and colocalization with the membrane (yellow, scale: 20 µm) (c) Orthogonal projections in the X-, Y-, and Z-planes (scale: 10 µm) further captured GL 20 transmembrane crossing

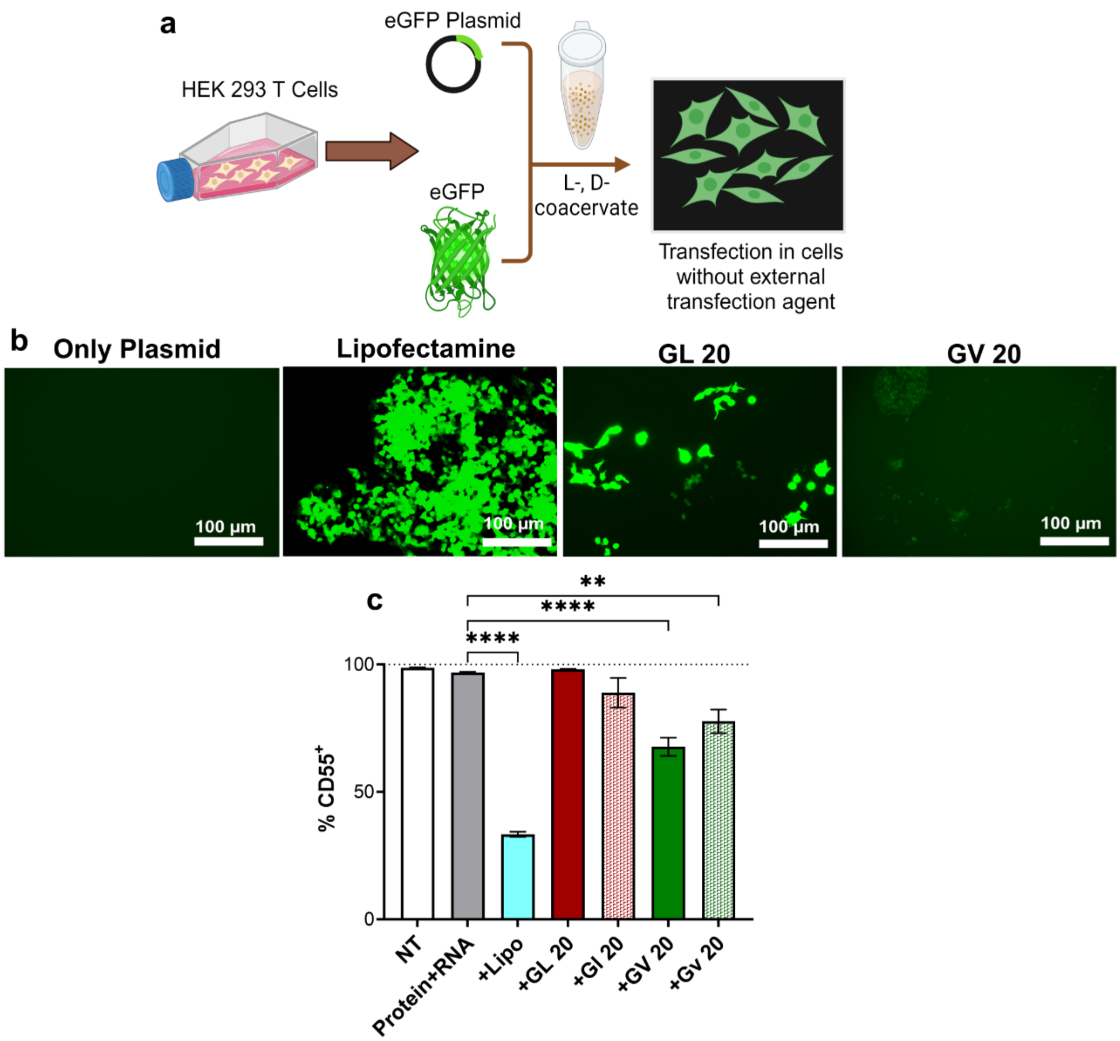

Figure S28. (a) Schematic showing the delivery of eGFP DNA plasmid. (b) Fluorescence micrographs of HEK293T cells transfected with (b) naked plasmid, lipofectamine-plasmid complexes or plasmid loaded in $(GHGLY)_4$ or $(GHGVY)_4$ coacervates. Images were taken 96 h after transfection. (c) CRISPR-Cas assay to determine knockdown of CD55 protein inside A549 cells using chiral $(GHGXY)_4$ (X=L/V).

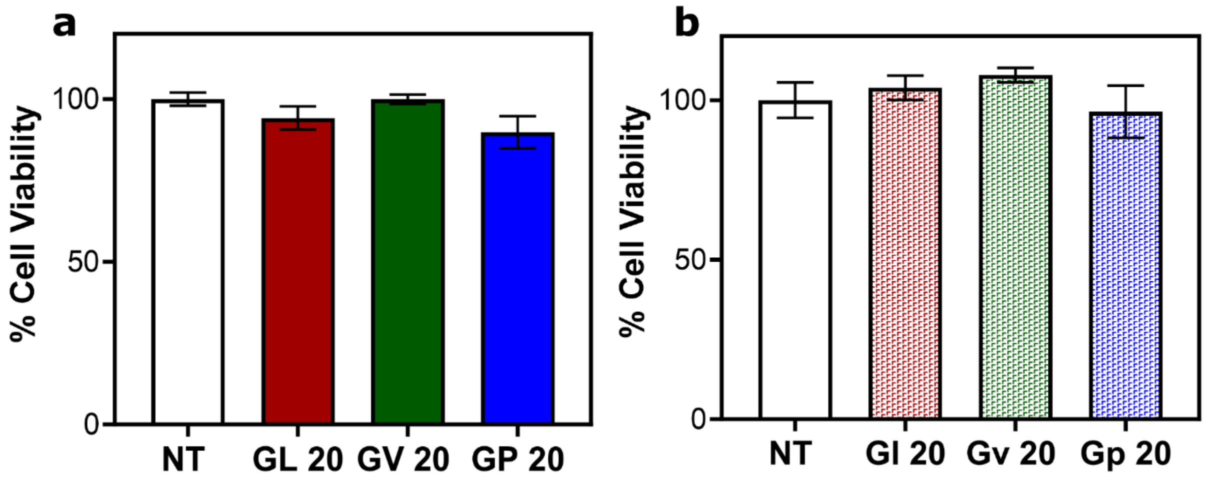

Figure S29. Viability of HEK293T cells following treatment with (a) L- or (b) D- $(GHGXY)_4$ coacervates (100 μM) for 24 h.

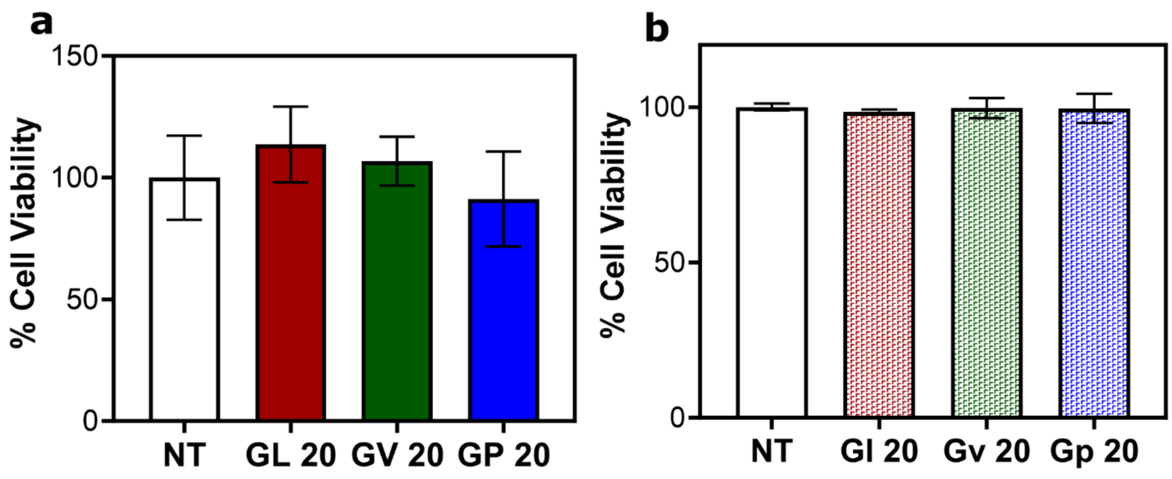

Figure S30. Viability of primary murine BMDCs following treatment with (a) L- or (b) D- $(GHGXY)_4$ coacervates (100 μM) for 24 h.

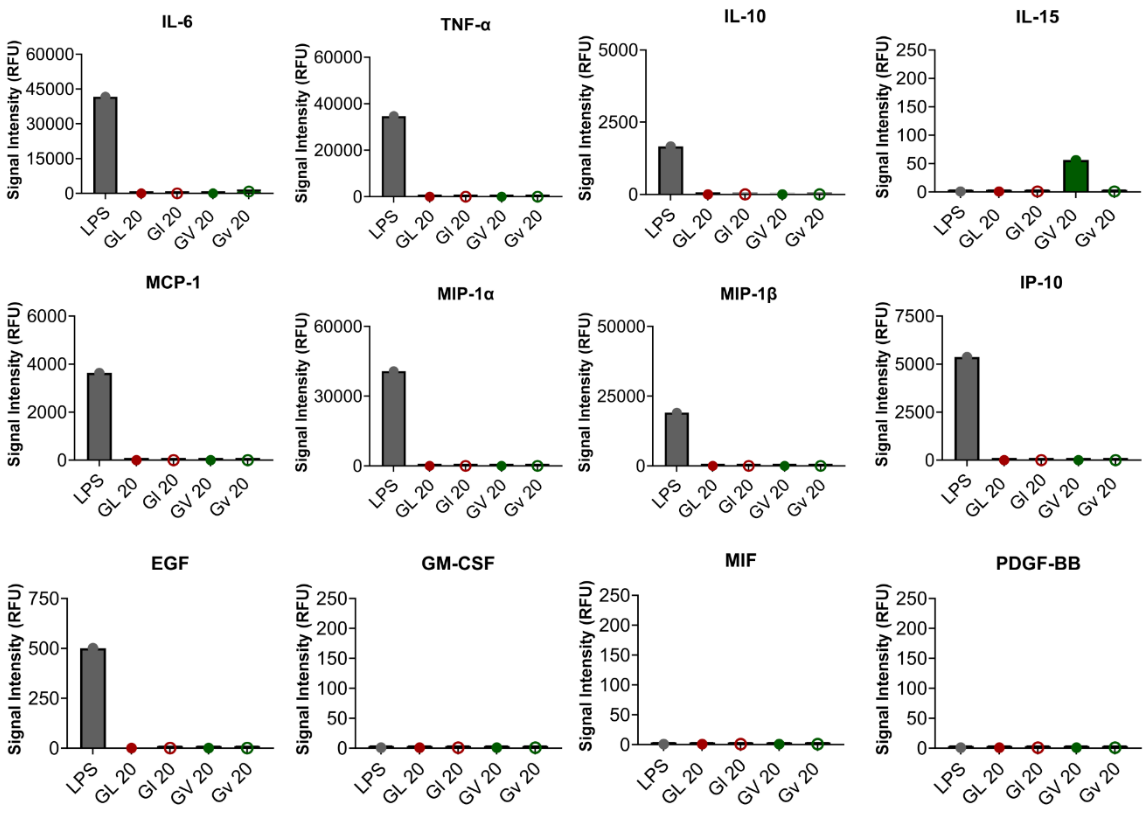

Figure S31. Cytokine (top row), chemokine (middle row) and (bottom row) growth factor production in BMDCs treated with L- or D- $(GHGXY)_4$ coacervates (10 µM) for 24 h.

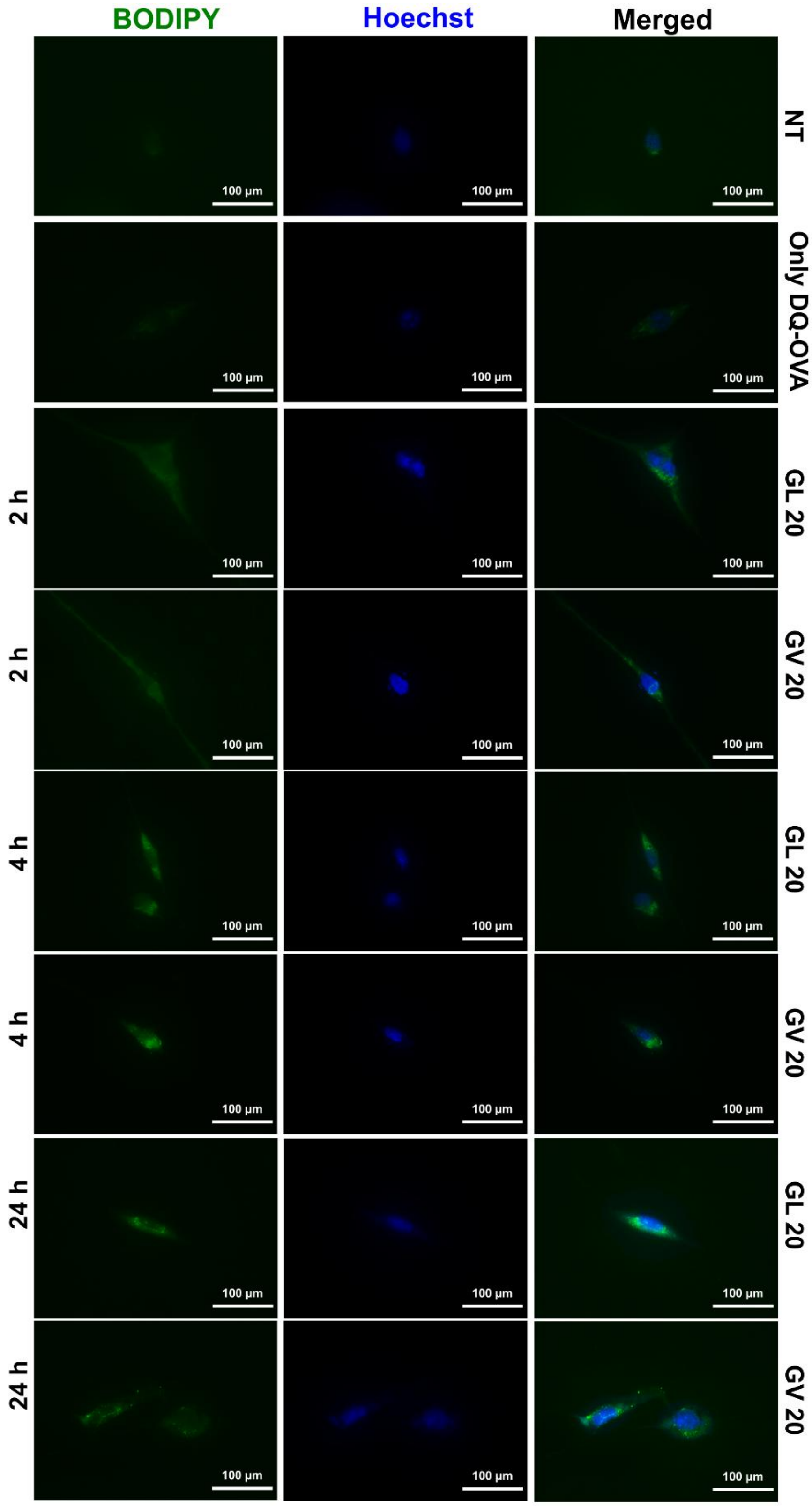

Figure S32. Fluorescence signal (green) from DQ-OVA processing in lysosomes following delivery with leucine or valine coacervates at different time points (2, 4, 24 h). Hoechst (blue) was used to stain the nucleus of BMDCs. Merged channels are also shown.

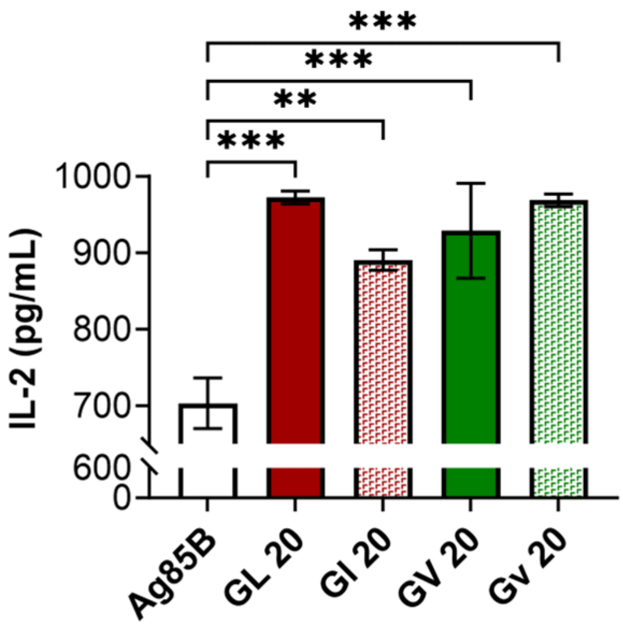

Figure S33. IL-2 is produced by BB7 hybridoma recognizing the processed Ag85B$_{240\text{-}254}$ epitope presented in the context of MHC II following delivery using leucine or valine coacervates. ****$p < 0.0001$ as determined by one-way ANOVA.

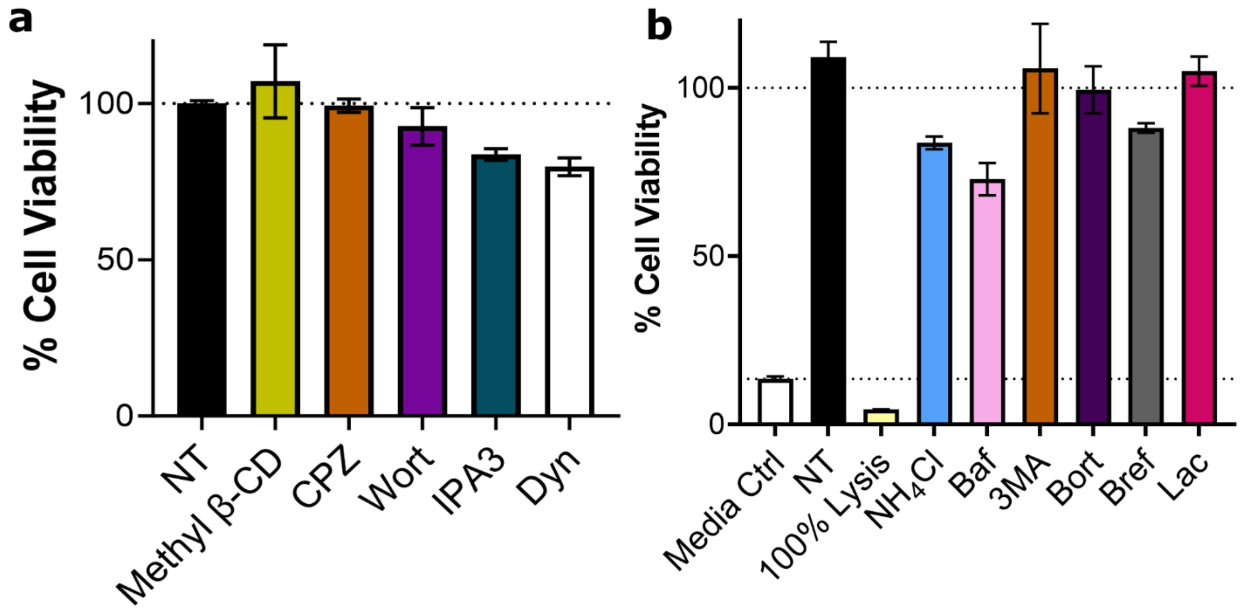

Figure S34. Viability of primary murine BMDCs following treatment with (a) endocytic pathway inhibitors (b) MHC class I and MHC class II pathway inhibitors at the concentrations used in the study.

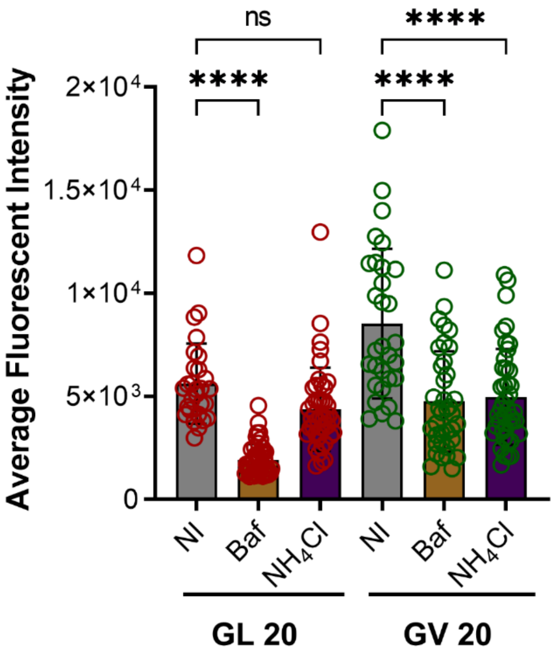

Figure S35. Reduction in fluorescence intensity of DQ-OVA delivered using leucine or valine coacervates in presence of Bafilomycin (Baf) or Ammonium Chloride (NH_4Cl). ****$p < 0.0001$ as determined by one-way ANOVA.

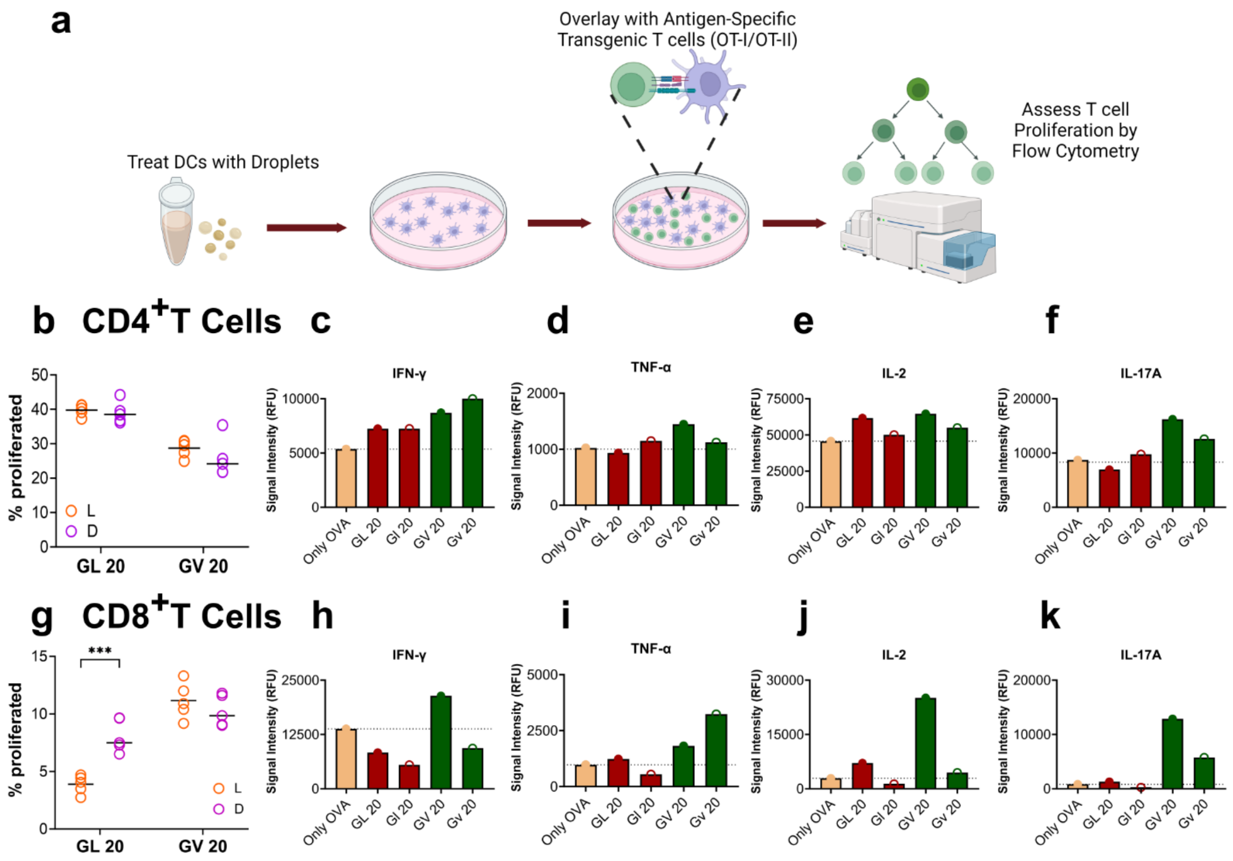

Figure S36. OT-I/OT-II mouse CD8⁺/CD4⁺T cell proliferation and cytokine release upon L- and D-(GHGXY)$_4$ (L/V) mediated delivery of OVA. (a) Schematic representation of antigen specific transgenic T cells (OT-I/OT-II mice) on antigen presenting mouse BMDCs to assess T cell proliferation. (b) OT-II mice CD4$^+$T and (g) OT-I mouse CD8$^+$T cell proliferation upon treatment with L- or D- (GHGXY)$_4$ coacervates loaded with OVA compared to control Data are shown as mean ± SEM of 5 technical replicates. Effect of L- and D- (GHGXY)$_4$ peptide coacervate-mediated OVA delivery on production of IFN-γ, TNF-α, IL-2, and IL-17A. Mouse BMDCs were treated with free OVA or coacervate-loaded OVA (10 µM) for 24 h before overlaying with CTV-labeled OT-II/OT-I hybridoma cells for 66 h followed by measurement of (c, h) IFN-γ, (d, i) TNF-α, (e, j) IL-2, (f, k) IL-17A in the culture supernatant of OT-II and OT-I mice, respectively. Values are shown as means of 2 wells in a Mouse Adaptive CodePlex Secretome chip pooled from treatments of 4 wells. ***p< 0.001, as determined by 2-way ANOVA analysis.

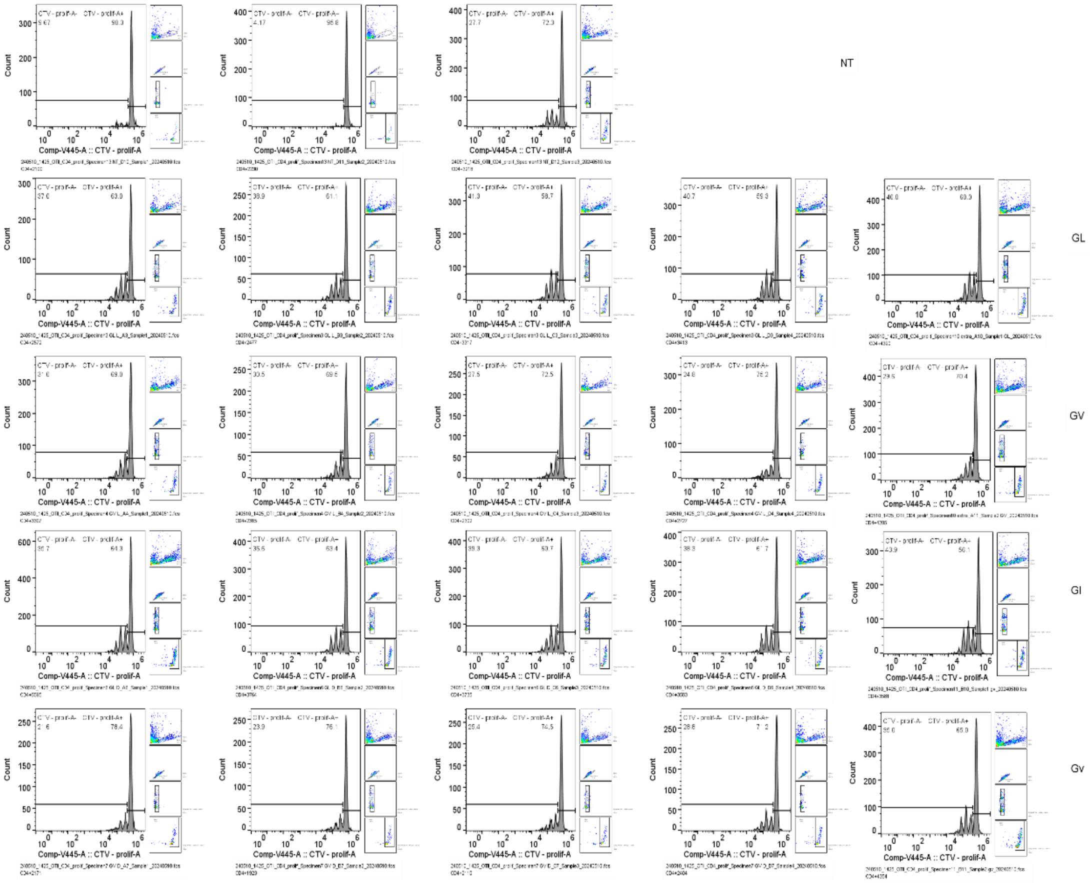

Figure S37. Individual histograms of CD4^{+}T-cell proliferation. CD4^{+}T-cells isolated from OT-II mice and labeled with CTV were overlaid on BMDCs treated with chiral $(GHGLY)_4$ or $(GHGVY)_4$ coacervates and proliferation measured as loss of CTV signal.

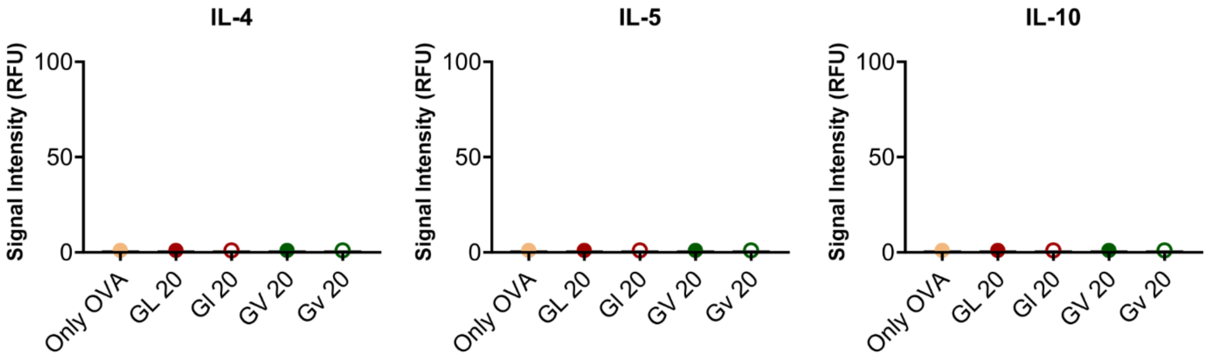

Figure S38. Adaptive immune responses depicting various levels of cytokines released upon treating OVA loaded L- and D- $(GHGXY)_4$ droplets with antigen presenting cells (mouse BMDCs) for 24 h and overlaying with CTV-labelled OT-II mice cells for 66 h.

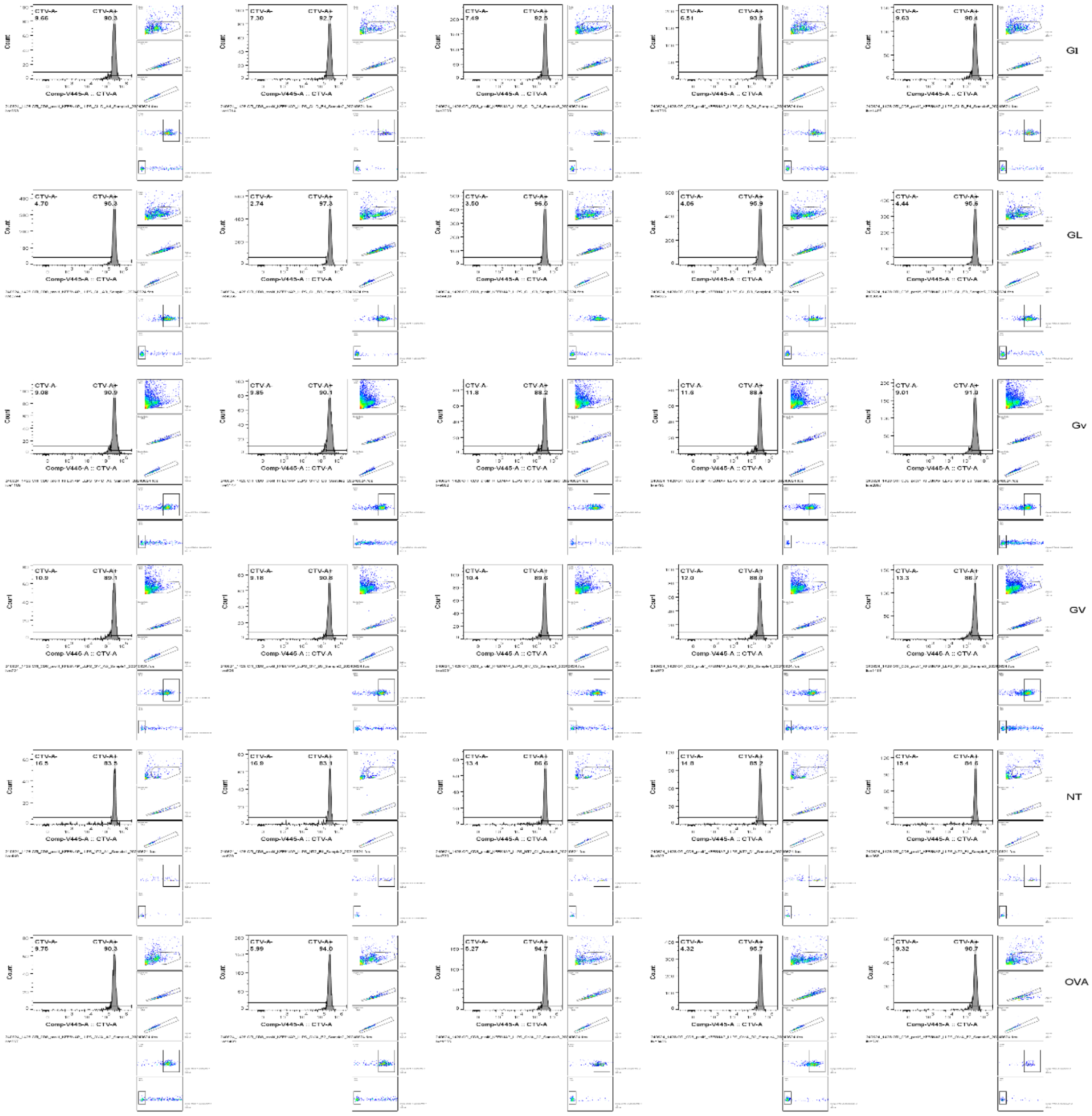

Figure S39. Individual histograms of CD8$^+$T-cell proliferation. CD8$^+$T-cells isolated from OT-I mice and labeled with CTV were overlaid on BMDCs treated with chiral $(GHGLY)_4$ or $(GHGVY)_4$ coacervates and proliferation measured as loss of CTV signal.

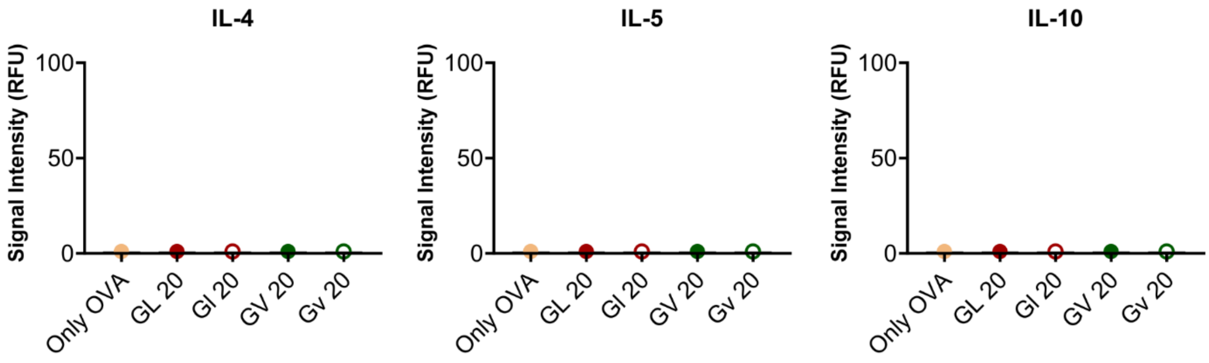

Figure S40. Adaptive immune responses depicting various levels of cytokines released upon treating OVA loaded L- and D- $(GHGXY)_4$ droplets with antigen presenting cells (mouse BMDCs) for 24 h and overlaying with CTV-labelled OT-I mice cells for 66 h.

REFERENCES

1 T. Lu, K. K. Nakashima and E. Spruijt, *J. Phys. Chem. B*, 2021, **125**, 3080–3091.

2 D. Van Der Spoel, E. Lindahl, B. Hess, G. Groenhof, A. E. Mark and H. J. C. Berendsen, *J. Comput. Chem.*, 2005, **26**, 1701–1718.

3 M. J. Abraham, T. Murtola, R. Schulz, S. Páll, J. C. Smith, B. Hess and E. Lindahl, *SoftwareX*, 2015, **1**, 19–25.

4 J. Huang, S. Rauscher, G. Nawrocki, T. Ran, M. Feig, B. L. de Groot, H. Grubmüller and A. D. MacKerell, *Nat. Methods*, 2017, **14**, 71–73.

5 J. Jumper, R. Evans, A. Pritzel, T. Green, M. Figurnov, O. Ronneberger, K. Tunyasuvunakool, R. Bates, A. Žídek, A. Potapenko, A. Bridgland, C. Meyer, S. A. A. Kohl, A. J. Ballard, A. Cowie, B. Romera-Paredes, S. Nikolov, R. Jain, J. Adler, T. Back, S. Petersen, D. Reiman, E. Clancy, M. Zielinski, M. Steinegger, M. Pacholska, T. Berghammer, S. Bodenstein, D. Silver, O. Vinyals, A. W. Senior, K. Kavukcuoglu, P. Kohli and D. Hassabis, *Nature*, 2021, **596**, 583–589.

6 S. Jo, T. Kim, V. G. Iyer and W. Im, *J. Comput. Chem.*, 2008, 29, 1859–1865.

7 A. Barth, *Biochim. Biophys. Acta* **-** *Bioenerg.*, 2007, **1767**, 1073–1101.

8 M. Jackson and H. H. Mantsch, *Crit. Rev. Biochem. Mol. Biol.*, 1995, **30**, 95–120.

9 A. Dong, P. Huang and W. S. Caughey, *Biochemistry*, 1990, **29**, 3303–3308.

10 X. Lian, C. Hsiao, G. Wilson, K. Zhu, L. B. Hazeltine, S. M. Azarin, K. K. Raval, J. Zhang, T. J. Kamp and S. P. Palecek, *Proc. Natl. Acad. Sci. U. S. A.*, 2012, **109**, E1848-57.

11 F. Lam, D. Cladière, C. Guillaume, K. Wassmann and S. Bolte, *Methods*, 2017, **115**, 17–27.

12 A. Baruch Leshem, S. Sloan-Dennison, T. Massarano, S. Ben-David, D. Graham, K. Faulds, H. E. Gottlieb, J. H. Chill and A. Lampel, *Nat. Commun.*, 2023, **14**, 421.

13 A. Khan, P. Bakhru, S. Saikolappan, K. Das, E. Soudani, C. R. Singh, J. L. Estrella, D. Zhang, C. Pasare, Y. Ma, J. Sun, J. Wang, R. L. Hunter, N. Tony Eissa, S. Dhandayuthapani and C. Jagannath, *npj Vaccines*, 2019, **4**, 34.

14 J. D. Pfeifer, M. J. Wick, R. L. Roberts, K. Findlay, S. J. Normark and C. V Harding, *Nature*, 1993, **361**, 359–362.

15 C. V. 3rd Harding, *Eur. J. Immunol.*, 1992, **22**, 1865–1869.

16 E. H. Noss, R. K. Pai, T. J. Sellati, J. D. Radolf, J. Belisle, D. T. Golenbock, W. H. Boom and C. V Harding, *J. Immunol.*, 2001, **167**, 910–918.
